# Accounting for Electronic Coherences Induced by Broadband Pulses by Using Pulse-Independent Trajectories

**DOI:** 10.1021/acs.jctc.5c01809

**Published:** 2026-01-20

**Authors:** Joachim Galiana, Stefano M. Cavaletto, Gilbert Grell, Francisco Fernández-Villoria, Alicia Palacios, Jesús González-Vázquez, Fernando Martín

**Affiliations:** † Departamento de Química, 16722 Universidad Autónoma de Madrid, Madrid 28049, Spain; ‡ 202533 Instituto Madrileño de Estudios Avanzados En Nanociencia (IMDEA Nanociencia), Madrid 28049, Spain; § Instituto de Física de la Materia Condensada (IFIMAC), 152669 Universidad Autónoma de Madrid, Madrid 28049, Spain

## Abstract

Recent advances in the generation of ultrashort, few-femtosecond laser pulses in the ultraviolet–visible domain are now enabling the coherent excitation of several electronic states in neutral molecules, with new opportunities for the manipulation of molecular dynamics on ultrafast time scales. Current time-resolved pump–probe experiments can monitor the ensuing coupled electron–nuclear dynamics with ultrashort resolution. Computational modeling of the observables measured in such experiments can be very challenging for medium-sized and large molecules because of (i) the nontrivial treatment of pump-generated coherences with mixed quantum–classical methods and (ii) the high computational cost of probe-step calculations, which cannot be afforded when many different pump pulses have to be considered, as e.g., in control schemes. In this work, we present two trajectory-surface-hopping approaches that include, *a posteriori,* the effect of the pump-generated coherences on the ensuing coupled electron–nuclear dynamics, thus avoiding performing a different coupled electron–nuclear dynamics calculation for every individual pump pulse. The effectiveness of both approaches is exemplified in glycine molecules excited by short ultraviolet pump pulses. We compare the results of both approaches with those obtained by including pump-generated coherences from the very beginning, showing an excellent agreement and confirming the important role of such initial coherences in the early nonadiabatic dynamics. Our results pave the way for both accurate and flexible simulations of pump–probe experiments or control studies in molecules excited by broadband laser sources.

## Graphical abstract

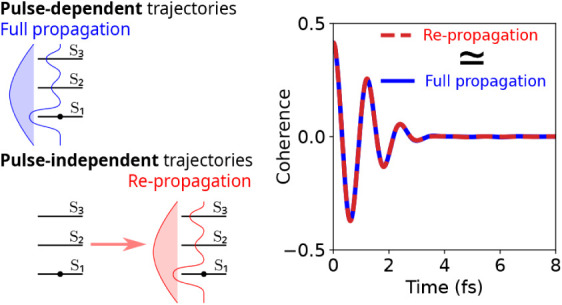

## 1 Introduction

The availability of attosecond extreme-ultraviolet (XUV) and X-ray pulses from sources based on high-order harmonic generation and free-electron lasers,^1–4^ and the recent generation of few-femtosecond pulses in the ultraviolet–visible (UV–vis) domain^5,6^ allow for the coherent excitation of several electronic states both in molecular cations^7–14^ and in neutral molecules.^15–17^ The coupled motion of electrons and nuclei following such a coherent excitation offers unprecedented opportunities for the control of photoinduced chemical reactions, a very active and timely line of research in the emerging field of attochemistry.^18–21^ In this regard, special emphasis is being placed in the optimization of intra- and intermolecular charge and energy transfers, and the design of photoswitches, with potential applications in photovoltaics.

The correct description of such initial coherent superpositions and, most importantly, their evolution in time accounting for the coupled motion of electrons and nuclei remain a challenge, in particular for medium-size and large organic molecules due to the large number of nuclear degrees of freedom.^22,23^ Full quantum dynamics methods are the natural choice for the treatment of electronic coherences, whose decay is dictated by the overlaps of the nuclear wave packets in the different electronic states. In this framework, well-established approaches, such as the multiconfigurational time-dependent Hartree (MCTDH)^24,25^ method and its Gaussian or multilayer (G-MCTDH and ML-MCTDH)^26–30^ extensions have been successfully applied to investigate the dynamics arising from an initial coherent superposition of electronic states of molecular cations, mimicking the conditions encountered when molecules are ionized by attosecond XUV or X-ray pulses, the only broadband pulses available until recently.^31–35^ However, these methods require parametrized potential energy surfaces, which can become prohibitively demanding when the number of nuclear degrees of freedom is large. A direct-dynamics variant of MCTDH, based on the variational multiconfigurational Gaussian (DD-vMCG) approach, has been put forward in order to evaluate the necessary energy derivatives on-the-fly without suffering from the parametrization of the potential energy surfaces.^36,37^ The DD-vMCG approach has been applied both in the context of coherent superpositions of cationic states^22,38^ and incoherent excited-state dynamics in neutral molecules.^39^ However, this strategy is computationally demanding for systematic studies with different pulses as it essentially relies on the propagation of coupled quantum trajectories.

Mixed quantum–classical methods, in which the nuclear degrees of freedom are treated classically, represent a more feasible alternative for large systems. Trajectory surface hopping (TSH) is one of the most widely implemented mixed quantum–classical approaches.^40,41^ In TSH, the initial nuclear wave packet is mimicked by a distribution of molecular geometries and momenta corresponding to a large set of initial conditions.^42–45^ A set of independent quantum–classical trajectories, one for each initial condition, is obtained by propagating the nuclear positions according to Newton’s equations of motions along the gradient of a single electronic potential, the so-called *active* state, whereas the corresponding electronic dynamics is described quantum mechanically according to the time-dependent Schrödinger equation (TDSE). The amplitudes of the electronic coefficients, obtained by solving the TDSE, are then employed at every time step to compute the probability for the trajectory to hop from the potential of the current active state to one of the inactive ones, due to the presence of nonadiabatic couplings. By classically treating the nuclei and not having to define nor propagate quantum nuclear wave packets, TSH can allow one to use highly accurate electronic structure methods and to explore several potential energy surfaces after photoexcitation without biases.

The original TSH approach intrinsically yields *overcoherences* because, for each trajectory, the electronic wave packet is defined at a single point in nuclear phase-space, which does not allow for gradually decreasing the overlap of nuclear wave packets evolving on different electronic potentials.^41,46–48^ This can be especially critical when approaching regions of strong nonadiabatic coupling, namely conical intersections (CoIns), where electronic coherences are generated.^49–52^ To improve the treatment of electronic decoherence, a number of variants of TSH have been proposed in the last decades,^53–59^ including the widely used energy-based decoherence correction (TSH-EDC). However, most of these TSH variants have focused on the description of decoherence when the individual trajectories approach a CoIn, and are not designed to model initial coherences generated by broadband pulses, which could be damped too harshly. Only very recently, motivated by recent advances in the generation of broadband pulses, trajectory-based approaches have been put forward to account for including pulse-induced electronic coherences. On the one hand, coupled-trajectory approaches were demonstrated for one-dimensional systems.^23,60^ On the other hand, independent-trajectory approaches, with decoherence corrections based on auxiliary quantities that are propagated along all electronic potentials, have been developed as extensions of the original TSH formulation, benefiting from its advantages regarding the system size.^61–67^ Among them, we highlight the recently proposed projected forces and momenta (TSH-PFM) decoherence correction,^67^ a further development in full dimensionality of a concept introduced by Jasper and Truhlar.^68^ In TSH-PFM, the respective auxiliary quantities are the inactive- and active-potential forces and momenta projected onto the direction of the velocity vector. In contrast to the full force vectors, the trajectory-velocity projected force can be obtained without evaluating additional inactive-potential gradients, hence within reasonable computational cost compared to the traditional TSH. TSH-PFM was shown to allow initial coherences to persist for time scales comparable to the quantum mechanical case, without suffering from overcoherence artifacts. The long-term behavior, after the decay of the initial coherence, was shown to be similar to that obtained with the well-established TSH-EDC for several molecules.^67^

In all trajectory-based approaches presented above, the nuclear and electronic degrees of freedom are simultaneously propagated from initial conditions that are directly determined by the properties of the pulse, including the details of the pump-generated coherences (PGCs). This represents a bottleneck when simulating the evolution of experimentally accessible observables, e.g., for a direct comparison with pump–probe experiments in which the properties of the pump may be varied upon optimizing the experimental setups, or for quantum control of the early electronic dynamics based on shaping the properties of the pump pulse. In all such cases, predicting the evolution of a physical observable requires computationally expensive single-point calculations, repeated for all nuclear configurations, i.e., for all initial conditions and electronic states involved in the coherent superposition at all time steps, which can easily amount to hundreds of thousands or millions of single-point calculations. When the physical observable is a photoelectron spectrum, which requires the evaluation of the electronic continuum, the computational effort is almost at the limit of what current computer resources can afford. A simulation strategy in which the evolution of the nuclear coordinates is determined by the properties of the pump pulse then implies that all these computationally expensive calculations need to be repeated whenever the pump pulse is modified. This is not only impractical, but it can render the systematic simulation of pump–probe experiments and quantum-control pulse-shaping schemes completely unfeasible, already for small to medium-sized organic molecules. A more flexible and applicable methodology, relying on “universal” trajectories that do not require repeating single-point calculations for any new set of pump-determined initial conditions, would thus be advisable.

Here, we propose two alternative approaches to include the PGCs in a postprocessing manner. In both approaches, a set of TSH simulations is first performed without previous knowledge of the initial pump pulse characteristics. This provides a set of preexisting, “universal” nuclear trajectories, that can be used to calculate the computationally expensive matrix elements necessary for the simulation of the physical observables. The role of the pump pulse and the associated PGCs are only included a posteriori, by repropagating the electronic TDSE along the set of previously obtained, frozen, nuclear trajectories, at almost no additional computational cost. We apply this theoretical framework to investigate the nonadiabatic dynamics of aligned, neutral glycine molecules, following coherent excitation by a linearly polarized UV pump pulse. Different pulse characteristics are considered, allowing for the excitation of superpositions of two and three electronic excited states. Decoherence is accounted for by using the recently proposed TSH-PFM method for the electronic dynamics. Results in which the PGCs are included a posteriori are compared with results in which the PGCs are included from the start in the propagation of the coupled electron–nuclear dynamics. This comparison confirms that the proposed postprocessing approaches correctly describe the evolution of key molecular properties and observables, such as populations, coherences, and dipoles; and that they are especially suitable to evaluate the early coherence dynamics within the first tens of femtoseconds, i.e., the temporal window set by the decay of the initial PGCs.

The paper is organized as follows. In Section 2, we present the different approaches to account for the PGCs, including them either from the start of the simulation, or at the postprocessing stage. After presenting the traditional TSH formalism, we describe the recently introduced TSH-PFM variant as originally designed to fully account for initial PGCs from the start of the coupled electron–nuclear dynamics simulation. We then show how one can repropagate the electronic dynamics along precomputed nuclear trajectories, a key requirement for including PGCs at the postprocessing stage of the coupled electron–nuclear dynamics simulation. Finally, we extend traditional independent-trajectory TSH approaches with this postprocessing step accounting for initial PGCs and yielding a more flexible and less computationally expensive protocol with respect to the use of different initial pump pulses. In Section 3, we illustrate the performance of these approaches by considering initial coherent superpositions of two and three excited electronic states of glycine generated by realistic ultrashort UV pulses. We show how initial coherences survive during the first few femtoseconds of the nonadiabatic dynamics and highlight their contribution to the molecular dipoles. The good agreement between PGCs included from the start and PGCs postprocessed, and the underlying reasons for such an agreement, are finally discussed. Conclusions are outlined in Section 4.

## 2 Theory and Methods

### 2.1 Trajectory Surface Hopping Simulations

The coupled electron–nuclear dynamics will be calculated within the trajectory-surface-hopping (TSH) framework. TSH is a mixed quantum–classical approach in which the coupled electron–nuclear dynamics is simulated by averaging over a set of *N*_t_ independent quantum–classical trajectories, here labeled by the index *j* ∈ {1, ..., *N*_t_}. For each quantum–classical trajectory, only the electronic degrees of freedom are propagated quantum mechanically according to the time-dependent Schrödinger equation (TDSE), whereas the nuclear degrees of freedom are propagated classically following Newton’s equations of motion. This is achieved by imposing that at every time step, the nuclear dynamics follows the gradient of a single electronic potential, referred to as the active potential *b* ^(*j*)^(*t*). In order to account for the nonadiabatic couplings and associated transitions, a stochastic algorithm is used to let trajectories hop from the active potential to one of the other potentials, so-called inactive potentials, with a probability depending on the electronic coefficients and populations. For a set of initial conditions given by the nuclear positions ***R*** ^(*j*)^(0), nuclear momenta ***P*** ^(*j*)^(0), electronic amplitudes ${\mathrm{c̃}}_{i}^{\left(j\right)}\left(0\right)$ , and initial active potential *b* ^(*j*)^(0), the resulting molecular wave packet is then given by

1 $$\left|{\mathrm{\Psi ̃}}^{\left(j\right)}\left(t\right)\right\rangle =\overset{N_{s}-1}{\underset{i=0}{\sum }}{\mathrm{c̃}}_{i}^{\left(j\right)}\left(t\right)\left|\psi_{i};R^{\left(j\right)}\left(t\right)\right\rangle$$

in which ***R*** ^(*j*)^(*t*) represents the evolution of the nuclear coordinates for the *j*th trajectory following the gradients of the active potential *b* ^(*j*)^(*t*), |ψ_*i*_; ***R*** ^(*j*)^(*t*)⟩ are the *N*_s_ considered adiabatic electronic states at the corresponding position in nuclear space, and ${\mathrm{c̃}}_{i}^{\left(j\right)}$ are the associated quantum amplitudes. The latter are then used to evaluate the probability to hop from the current active potential *b* ^(*j*)^(*t*) to one of the inactive potentials. This in turn determines the subsequent evolution of the nuclear coordinates ***R*** ^(*j*)^(*t*), which are obtained by integrating classical Newton’s equations of motion with gradients computed in the active potential *b* ^(*j*)^(*t*). Atomic units are used in the following, unless otherwise specified.

The set of initial nuclear positions and momenta for the *N*_t_ trajectories can be set according to a Wigner distribution, which allows one to mimic and reproduce the properties of an initial nuclear wave packet. This is often referred to as a nuclear ensemble approach in the realm of mixed quantum–classical methods, such as TSH.^42,43^ Different strategies for the initialization of the electronic coefficients ${\mathrm{c̃}}_{i}^{\left(j\right)}\left(0\right)$ , especially in the presence of initial pulse-generated coherences (PGCs), will be discussed in the following. An ensemble of TSH trajectories is then considered internally consistent,^40,41,53^ if the average of the population of the *i*th electronic state is equal to the fraction of trajectories *j* with active potential *b* ^(*j*)^(*t*) = *i*, i.e.,

2 $$\frac{1}{N_{t}}\overset{N_{t}}{\underset{j=1}{\sum }}{|{\mathrm{c̃}}_{i}^{\left(j\right)}\left(t\right)|}^{2}≃\frac{1}{N_{t}}\overset{N_{t}}{\underset{j=1}{\sum }}\delta_{ib^{\left(j\right)}\left(t\right)}$$

Ensuring internal consistency within the original TSH formulation can be hindered by the fact that TSH does not include any mechanisms mimicking the quantum character of nuclear wave packets on different electronic potential energy surfaces. A number of approximations and decoherence corrections have been implemented in order to address this issue. These decoherence corrections consist in including, already at the single-trajectory level, a decay mechanism for the coherences ${\mathrm{\rho ̃}}_{i^{\prime }i}^{\left(j\right)}\left(t\right)={[{\mathrm{c̃}}_{i}^{\left(j\right)}\left(t\right)]}^{*}{\mathrm{c̃}}_{i^{\prime }}^{\left(j\right)}\left(t\right)$ .^53–59^ Among them, we highlight the widely used energy-based decoherence correction (TSH-EDC) proposed by Granucci and Persico inspired by Zhu and Truhlar original work,^54–56^ which has been designed to estimate decoherence around points of energy degeneracy such as CoIns of the potential energy surfaces.

In analogy to eq 2, the expectation value of a generic operator *Â* is estimated in TSH through a simple average exclusively involving the active-state wave functions,

3 $$\left\langle {Â}_{\mathrm{TSH}}\left(t\right)\right\rangle ≃\frac{1}{N_{t}}\overset{N_{t}}{\underset{j=1}{\sum }}\left\langle \psi_{b^{\left(j\right)}\left(t\right)};R^{\left(j\right)}\left(t\right)\left|Â\right|\psi_{b^{\left(j\right)}\left(t\right)};R^{\left(j\right)}\left(t\right)\right\rangle$$

Note that eq 3 disregards both the adiabatic electronic populations |*c̃*_*i*_^(*j*)^(*t*)|^2^ and coherences ${[{\mathrm{c̃}}_{i}^{\left(j\right)}\left(t\right)]}^{*}{\mathrm{c̃}}_{i^{\prime }}^{\left(j\right)}\left(t\right)$ in the evaluation of the observables. This is justified when internal consistency is satisfied and electronic coherences are small, rendering this approach usable for modeling photoinduced dynamics in the context of femtochemistry. However, the insensitivity of eq 3 to electronic coherences renders it not suitable to describe nonadiabatic dynamics excited by ultrashort pulses where coherent superpositions of states are generated. In such scenario, the inclusion of initial PGCs and their decay is essential both for the ensemble of trajectories and on the individual-trajectory level, where wave function information from both active and inactive states is required, as will be highlighted in Section 2.2. Hereafter, we will thus employ the recently proposed projected forces and momenta decoherence correction (TSH-PFM),^67^ which has been designed to accurately account for both (i) initial coherences generated by ultrashort pulses, and (ii) coherences induced by the passage through a CoIn, within the inherent limitations of TSH due to the classical propagation of the nuclei. In the following sections, we discuss how to fully account for electronic coherence with TSH simulations corrected with the PFM decoherence approach.

### 2.2 Full Propagation of Coherences in TSH Simulations

In this section, we present our protocol for the full propagation (FP) of electron and nuclear dynamics in the presence of initial PGCs with the recently introduced TSH-PFM methodology.^67^ Due to the presence of PGCs, we assume that the quantum state of the *j*th trajectory in eq 1 at *t* = 0 is given by the following coherent superposition state

4 $$\left|{\mathrm{\Psi ̃}}^{\left(j\right)}\left(0\right)\right\rangle =\overset{N_{s}-1}{\underset{i=0}{\sum }}{\mathrm{c̃}}_{i}^{\left(j\right)}\left(0\right)\left|\psi_{i};R^{\left(j\right)}\left(0\right)\right\rangle$$

As for traditional TSH, also in TSH-PFM the evolution of the electronic coefficients ${\mathrm{c̃}}_{i}^{\left(j\right)}\left(t\right)$ is obtained by integrating the time-dependent Schrödinger equation

5 $$i\frac{d{\mathrm{c̃}}_{k}^{\left(j\right)}\left(t\right)}{dt}=\overset{N_{s}-1}{\underset{i=0}{\sum }}\left[V_{ki}\left(R^{\left(j\right)}\left(t\right)\right)-iT_{ki}\left(R^{\left(j\right)}\left(t\right)\right)\right]{\mathrm{c̃}}_{i}^{\left(j\right)}\left(t\right)$$

where *V*_*ki*_(***R*** ^(*j*)^(*t*)) is the matrix element of the electronic Hamiltonian for states *i* and *k* and *T*_*ki*_(***R*** ^(*j*)^(*t*)) is the time-derivative coupling between states *k* and *i*, both evaluated at the nuclear geometry ***R*** ^(*j*)^(*t*). In practice, we numerically integrate this equation in a local diabatic basis and transform back the coefficients to the adiabatic basis, see Supporting Information of ref 67. However, in contrast to traditional TSH, in TSH-PFM as well as in other decoherence-corrected TSH approaches, the adiabatic electronic coefficients are corrected with a decoherence rate that takes into account the projected forces and momenta, hence including information from auxiliary quantities propagated in the inactive potentials that have nonzero populations. The nuclear coordinates ***R*** ^(*j*)^(*t*) and the amplitudes ${\mathrm{c̃}}_{i}^{\left(j\right)}\left(t\right)$ are then propagated simultaneously, accounting for the initial pulse-generated coherences (PGCs).

When averaging over a sufficiently large ensemble of trajectories, TSH-PFM has been shown to provide a suitable description for the evolution of the electronic coherences, ${\mathrm{\rho ̃}}_{i^{\prime }i}^{\left(j\right)}\left(t\right)={[{\mathrm{c̃}}_{i}^{\left(j\right)}\left(t\right)]}^{*}{\mathrm{c̃}}_{i^{\prime }}^{\left(j\right)}\left(t\right)$ .^67^ In contrast to eq 3, in which the expectation value of an operator ⟨*Â*⟩ is evaluated based exclusively on the evolution of the active state wave function, the TSH-PFM methodology renders the electronic coefficients ${\mathrm{c̃}}_{i}^{\left(j\right)}\left(t\right)$ , and thus the associated electronic populations and coherences, usable for the evaluation of ⟨*Â*⟩. Under these conditions, the expectation value of a generic operator *Â* can then be obtained by calculating the expectation values over the electronic wave functions and averaging them over the ensemble of trajectories

⟨Â(t)⟩=1Nt∑j=1Nt⟨Ψ̃(j)(t)|Â|Ψ̃(j)(t)⟩=1Nt∑j=1Nt∑i,i′=0Ns−1[c̃i(j)(t)]*c̃i′(j)(t)⟨ψi;R(j)(t)|Â|ψi′;R(j)(t)⟩ 6

Two contributions can be highlighted in eq 6: a first contribution related to populations

7 $$A_{\mathrm{pop}}\left(t\right)=\frac{1}{N_{t}}\overset{N_{t}}{\underset{j=1}{\sum }}\overset{N_{s}-1}{\underset{i=0}{\sum }}{|{\mathrm{c̃}}_{i}^{\left(j\right)}\left(t\right)|}^{2}\left\langle \psi_{i};R^{\left(j\right)}\left(t\right)\left|Â\right|\psi_{i};R^{\left(j\right)}\left(t\right)\right\rangle$$

and a second one to electronic coherences

$$\begin{array}{l} A_{\mathrm{coh}}\left(t\right)=\frac{1}{N_{t}}\overset{N_{t}}{\underset{j=1}{\sum }}\underset{i^{\prime }\neq i}{\overset{N_{s}-1}{\underset{i,i^{\prime }=0}{\sum }}}{[{\mathrm{c̃}}_{i}^{\left(j\right)}\left(t\right)]}^{*}{\mathrm{c̃}}_{i^{\prime }}^{\left(j\right)}\left(t\right)\left\langle \psi_{i};R^{\left(j\right)}\left(t\right)\left|Â\right|\psi_{i^{\prime }};R^{\left(j\right)}\left(t\right)\right\rangle \end{array}$$ 8

so that ⟨*Â*(*t*)⟩ = *A*_pop_(*t*) + *A*_coh_(*t*). As mentioned in Section 2.1, evaluating the expectation values according to eq 3 would completely disregard the coherence contribution *A*_coh_, while the population contribution *A*_pop_ would be reduced to the contribution from the active states only. In the remaining of the paper, the evolution of all observables will be obtained according to eq 6 which is sensitive to electronic coherences. Employing the ${\mathrm{c̃}}_{i}^{\left(j\right)}\left(t\right)$ in the evaluation of eq 6 can be justified by the fact that proper account for decoherence establishes internal consistency in TSH and ensures that observables obtained with eq 3 and eq 7 agree to a good approximation.^53,69^

To illustrate our theoretical framework in the presence of PGCs, in the following, we assume an ensemble of trajectories fully indexed by the label *j* = *g* ∈ {1, ..., *N*_g_}, where *N*_g_ is the number of geometries. The index *g* here labels the initial nuclear coordinates and momenta, ***R*** ^(*g*)^(0) and ***P*** ^(*g*)^(0), respectively, as well as the associated initial electronic coefficients $\left\{{\mathrm{c̃}}_{i}^{\left(g\right)}\left(0\right)\right\}$ and initial active potential *b* ^(*g*)^(0). For each geometry, the electronic coefficients are set according to the coherent superposition in eq 4 and irrespective of the initial active potential, *b* ^(*g*)^(0), which is instead determined stochastically. The probability to choose the *i*th electronic state as active is proportional to its initial population $|{\mathrm{c̃}}_{i}^{\left(g\right)}\left(0\right){|}^{2}$ . The framework is illustrated in Figure 1(a), where for each geometry *g*, only one active state is defined for the nuclear dynamics, but the initial coherences (blue solid line, representing the initial coherent superposition of states generated by the pulse, in the transparent blue shape) are included and accounted for in the electron dynamics. The expectation value ⟨*Â*(*t*)⟩ is evaluated using eq 6, which we now write for the ensemble of geometries indexed by *g*,

9 $$\left\langle Â\left(t\right)\right\rangle =\frac{1}{N_{g}}\overset{N_{g}}{\underset{g=1}{\sum }}\left\langle {\mathrm{\Psi ̃}}^{\left(g\right)}\left(t\right)\left|Â\right|{\mathrm{\Psi ̃}}^{\left(g\right)}\left(t\right)\right\rangle$$

where both contributions from populations and coherences are accounted for. In the following, we refer to this approach as PGCs included from the start.

**Figure 1:**
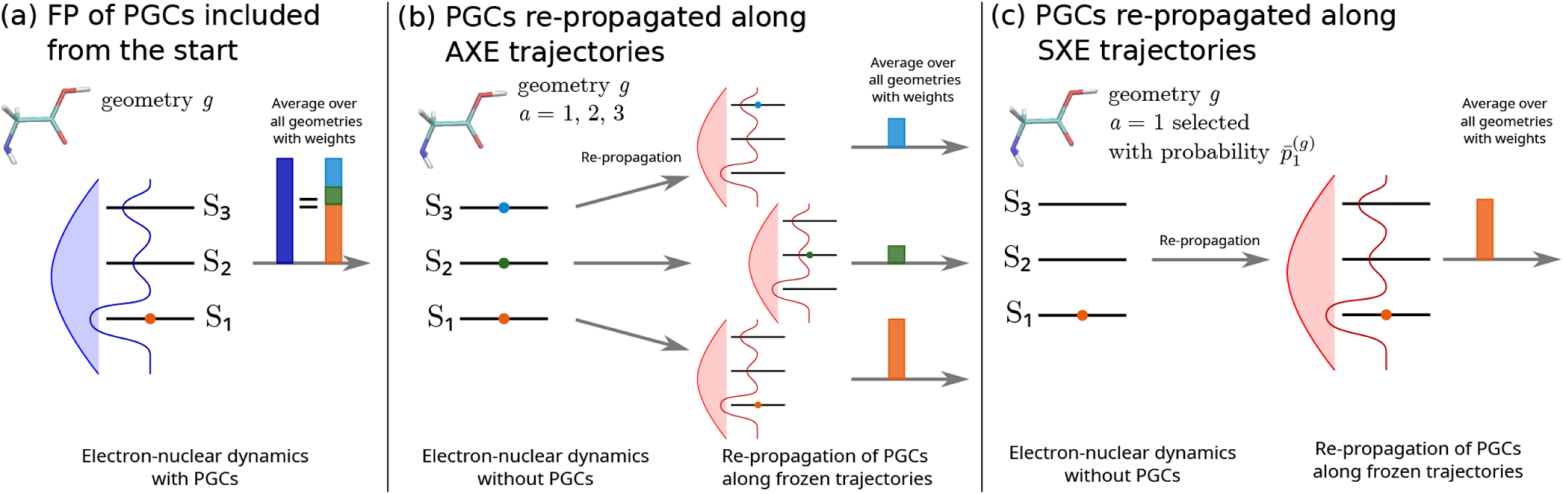
Schematic description of three trajectory-based methodologies starting from an initial coherent superposition of states, including the pump-generated coherences (PGCs). Panel (a): Full propagation (FP) where, for every geometry *g*, the PGCs are included from the start in the simulation of the coupled electron and nuclear dynamics. The blue bell represents the spectrum of the broadband pulse, and the blue solid line represents the corresponding absorption spectrum reflecting the initial excited electronic coefficients $c_{i}^{\left(g\right)}\left(0\right)$ . The average is performed over all geometries with geometry-dependent weights proportional to the total excited population (dark blue solid vertical bar), equal to the sum of the populations in the three excited states (orange, green, and blue solid vertical bars, respectively). Panels (b, c): For every initial condition, the PGCs are included by repropagating the electronic coefficients along precomputed nuclear dynamics from (b) the AXE trajectories and (c) the SXE trajectories, respectively. For the AXE in (b, left), for every geometry *g*, the trajectories are computed for all possible initial conditions *a* = 1, 2, 3 in the absence of PGCs, whereas for the SXE in (c, left), for every geometry *g*, the trajectories are computed only on the selected initial conditions (*a* = 1 in this example, see eq 16 and Appendix A for details) in the absence of PGCs. In both cases, the electron dynamics are then repropagated for each precomputed, frozen trajectory including the PGCs (b and c, right). In analogy to (a), the red bells represent the spectrum of the postprocessed broadband pulse, and the red solid lines represent the corresponding absorption spectrum reflecting the initial excited electronic coefficients $c_{i}^{\left(g\right)}\left(0\right)$ . The averages are performed over all geometries with geometry- and state-dependent weights that are proportional to the initial populations in the presence of the pulse and are depicted (b) by the orange, green, and blue solid vertical bars for the AXE trajectories (see eq 18) and (c) the orange vertical bar for the SXE trajectories (see eq 21). In all three cases, the orange, green, or blue dots represent the initial active potential S_1_, S_2_, or S_3_, respectively, for TSH.

We note for completeness that for studies of excited-state dynamics within the TSH formalism, it is customary to only propagate the excited-state part of the initial electronic wave function, not including the contribution due to the initial ground state amplitude ${\mathrm{c̃}}_{0}^{\left(j\right)}\left(0\right)$ . This corresponds to the propagation of the coherent superposition

10 $$\left|\Psi^{\left(g\right)}\left(0\right)\right\rangle =\overset{N_{s}-1}{\underset{i=1}{\sum }}c_{i}^{\left(g\right)}\left(0\right)\left|\psi_{i};R^{\left(g\right)}\left(0\right)\right\rangle$$

with newly defined initial amplitudes $c_{i}^{\left(g\right)}\left(0\right)$ related to the pump-generated ones ${\mathrm{c̃}}_{i}^{\left(g\right)}\left(0\right)$ via $c_{i}^{\left(g\right)}\left(0\right)={\mathrm{c̃}}_{i}^{\left(g\right)}\left(0\right)/\left(\sqrt{\overset{N_{s}-1}{\underset{k=1}{\sum }}{|{\mathrm{c̃}}_{k}^{\left(g\right)}\left(0\right)|}^{2}}\right)$ . The coefficients $c_{i}^{\left(g\right)}\left(0\right)$ are then propagated in time to yield the electronic coefficients $c_{i}^{\left(g\right)}\left(t\right)$ . In the following, only these expectation values, obtained by propagating the excited-state part of the initial electronic wave packet, are considered. The above normalization ensures that the sum of the populations in the excited states is equal to 1, i.e., $\overset{N_{s}-1}{\underset{k=1}{\sum }}|c_{k}^{\left(g\right)}\left(t\right){|}^{2}=1$ . Equation 9 can then be recast in terms of the normalized electronic wave function and the amplitudes as

⟨ÂFP(t)⟩=1Ng∑g=1Ng(∑k=1Ns−1|c̃k(g)(0)|2)⟨Ψ(g)(t)|Â|Ψ(g)(t)⟩=1Ng∑g=1Ng(∑k=1Ns−1|c̃k(g)(0)|2)∑i,i′=0Ns−1[ci(g)(t)]*ci′(g)(t)×⟨ψi;R(g)(t)|Â|ψi′;R(g)(t)⟩ 11

where the FP subscript stands for *full propagation* and reflects the fact that the nonadiabatic dynamics is propagated including the PGCs from the start of the simulation. Note that eq 11 has the structure of an average of individual-trajectory expectation values *A*_*g*_(*t*) = ⟨Ψ^(*g)*^(*t*)|*Â*|Ψ^(*g*)^(*t*)⟩, where the total absorption probability in the presence of the pulse acts as a geometry-dependent weight, $w_{g}=N_{g}^{-1}\overset{N_{s}-1}{\underset{k=1}{\sum }}|{\mathrm{c̃}}_{k}^{\left(g\right)}\left(0\right){|}^{2}$ .

Within the full-propagation approach described above, the coupled electron and nuclear dynamics fully depend on the coefficients ${\mathrm{c̃}}_{i}^{\left(g\right)}\left(0\right)$ of the electronic wave packet of the initial coherent superposition. This leads to the possible issue that each trajectory ***R*** ^(*g*)^(*t*) and therefore all the relevant matrix elements ⟨ψ_*i*_; ***R*** ^(*g*)^(*t*)|*Â*|ψ_*i’*_; ***R*** ^(*g*)^(*t*)⟩ completely depend on the initial conditions ${\mathrm{c̃}}_{i}^{\left(g\right)}\left(0\right)$ determined by the pump pulse. Varying the properties of the pump excitation thus implies that all trajectories and relevant matrix elements need to be recomputed. This can represent a challenge when the computationally expensive matrix elements are required for the evaluation of signals, for instance when simulating time-resolved photoelectron spectra.

### 2.3 Repropagation of Coherences in TSH Simulations

To address this issue, we hereby propose an alternative treatment of the initial electronic coefficients. Let us assume that the coupled electron–nuclear dynamics has been propagated once for the initial condition *j*, $\left\{R^{\left(j\right)}\left(0\right),P^{\left(j\right)}\left(0\right),\left\{{\mathrm{c̃}}_{i}^{\left(j\right)}\left(0\right)\right\},b^{\left(j\right)}\left(0\right)\right\}$ . In conventional TSH (Section 2.1), the initial electronic coefficients are given by ${\mathrm{c̃}}_{i}^{\left(j\right)}\left(0\right)=\delta_{ib^{\left(j\right)}\left(0\right)}$ , while in the previously described full-propagation approach (Section 2.2), they are defined by the normalized coefficients of the initial coherent superposition given by eq 4. In both cases, the resulting trajectory ***R*** ^(*j*)^(*t*) is determined by the evolution of the active potential *b* ^(*j*)^(*t*) which ultimately depends on the choice of the initial electronic coefficients, $\left\{{\mathrm{c̃}}_{i}^{\left(j\right)}\left(0\right)\right\}$ . If one starts from a slightly different initial condition, varying only the initial electronic coefficients, $\left\{R^{\left(j\right)}\left(0\right),P^{\left(j\right)}\left(0\right),\left\{{\mathrm{c̃}}_{i}^{\prime \left(j\right)}\left(0\right)\right\},b^{\left(j\right)}\left(0\right)\right\}$ , the full propagation of the equations of motion will yield new electronic coefficients *c̃*′^(*j*)^(*t*) leading to a different evolution of the active potential *b*′^(*j*)^(*t*) and, thus, a different trajectory ***R*** ′^(*j*)^(*t*). To avoid repeating the calculation of the trajectory ***R*** ′^(*j*)^(*t*), and the associated computationally expensive properties, we propose to only repropagate the electronic dynamics by integrating the TDSE along the trajectory ***R*** ^(*j*)^(*t*) previously computed for *b* ^(*j*)^(*t*), thus obtaining new electronic coefficients ${\mathrm{C̃}}_{i}^{\left(j\right)}$ given by the solution of

$$i\frac{d{\mathrm{C̃}}_{k}^{\left(j\right)}\left(t\right)}{dt}=\overset{N_{s}-1}{\underset{i=0}{\sum }}\left[V_{ki}\left(R^{\left(j\right)}\left(t\right)\right)-iT_{ki}\left(R^{\left(j\right)}\left(t\right)\right)\right]{\mathrm{C̃}}_{i}^{\left(j\right)}\left(t\right)$$ 12

which is, in practice, numerically integrated in a local diabatic basis. Equation 12 differs from eq 5 in the fact that the geometry-dependent quantities are taken from the precomputed trajectories ***R*** ^(*j*)^(*t*) rather than from the ***R*** ′^(*j*)^ ones, which we avoid calculating. This *repropagation* (RP) of the electronic dynamics is exact if there are no hops, e.g., in a Born–Oppenheimer dynamics where the trajectories ***R*** ^(*j*)^(*t*) and ***R*** ′^(*j*)^(*t*) follow the gradient of the same active potential throughout the whole simulation. In such limiting case, the evolution of the electronic coefficients ${\mathrm{C̃}}_{i}^{\left(j\right)}\left(t\right)$ still depends, through eq 12, on the evolution of the nuclear geometry ***R*** ^(*j*)^(*t*). However, because of the absence of hops, ***R*** ^(*j*)^(*t*) is not dependent on the electronic coefficients and does not need to be recomputed. Although this repropagation strategy is exact in the absence of hops, we aim at applying it in the more general context of TSH, where the active potential can vary in time. If the active potential *b* ^(*j*)^(*t*) and the nuclear trajectory ***R*** ^(*j*)^(*t*) do not depend strongly on the change of the initial electronic coefficients, the new electronic coefficients ${\mathrm{C̃}}_{i}^{\left(j\right)}\left(t\right)$ , obtained by repropagating the electronic dynamics along the precomputed trajectories ***R*** ^(*j*)^(*t*), can then be assumed to be a good approximation of the coefficients ${\mathrm{c̃}}_{i}^{\prime \left(j\right)}\left(t\right)$ that one would obtain with the full propagation of the coupled electron–nuclear dynamics. Under these conditions, any choice of initial electronic coefficients would be suitable to compute the “universal” (with respect to the initial excitation) trajectories ***R*** ^(*j*)^(*t*). In the following, we will obtain the trajectories ***R*** ^(*j*)^(*t*) for the particular case in which no initial coherences are present, ${\mathrm{c̃}}_{i}^{\left(j\right)}\left(0\right)=\delta_{ib^{\left(j\right)}\left(0\right)}$ . The trajectories thereby obtained will then act as a support for the repropagation of the electronic coefficients, allowing for the inclusion of PGCs a posteriori, at almost no additional cost.

This repropagation approach of the electronic dynamics along precomputed nuclear trajectories relies on the underlying approximation that the early nuclear dynamics is not or only slightly affected by the initial electronic coherences. In this work, we aim to numerically verify the validity of this approach, showing how it can be properly implemented for the evaluation of the expectation values, and their evolution in time, over an ensemble of initial geometries. Toward this goal, in the following sections, we first revise different nuclear ensemble approaches for the evaluation of expectation values in the absence of PGCs, to then generalize and implement them in the presence of initial electronic coherences with repropagated electronic dynamics along pulse-independent trajectories.

### 2.4 Pulse-Independent Trajectories in the Absence of Initial Coherences

In this section, we revise ensemble approaches for the evaluation of dynamic observables in the absence of initial coherences.^43,44,70^ We first consider a set of initial conditions for TSH simulations *j* = (*g*,*a*), labeled by a couple of indices: *g* running over a distribution of *N*_g_ initial geometries and, for each *g*, an index *a* running over all possible (*N*_s_ – 1) excited states. Herein and from now on, we refer to this as the All eXcited-state Ensemble (AXE) and the corresponding trajectories are referred to as the AXE trajectories. For every initial geometry ***R*** ^(*g*)^(0), one computes (*N*_s_ – 1) trajectories and associated wave functions

13 $$\left|\Psi^{\left(g,a\right)}\left(t\right)\right\rangle =\overset{N_{s}-1}{\underset{i=0}{\sum }}c_{i}^{\left(g,a\right)}\left(t\right)\left|\psi_{i};R^{\left(g,a\right)}\left(t\right)\right\rangle$$

one for each electronic excited state *a* ∈ {1, ..., *N*_s_ – 1}. For every trajectory, the initial amplitudes at *t* = 0 are set as $c_{i}^{\left(g,a\right)}\left(0\right)=\delta_{ia}$ , thus assuming that the total population at *t* = 0 is in the electronic state *a*,

14 $$\left|\Psi^{\left(g,a\right)}\left(0\right)\right\rangle =\left|\psi_{a};R^{\left(g\right)}\left(0\right)\right\rangle$$

We note here that ***R*** ^(*g*,*a*)^(0) = ***R*** ^(*g*)^(0) for all active states *a* for a given initial geometry *g*, but that they differ at all other times, hence the systematic use of the superscript (*g*,*a*) for *t* > 0. Equation 14 implies that the individual trajectories do not account for the actual initial populations $|{\mathrm{c̃}}_{a}^{\left(g\right)}\left(0\right){|}^{2}$ , see eq 4, which are excited by the pump pulse into the different excited states. The effect of these initial populations can be included when computing the expectation values as an average over all *N*_g_ × (*N*_s_ – 1) trajectories, weighted by the corresponding pump-generated populations $|{\mathrm{c̃}}_{a}^{\left(g\right)}\left(0\right){|}^{2}$ ,

15 $$\left\langle {Â}_{\mathrm{AXE}}\left(t\right)\right\rangle =\frac{1}{N_{g}}\overset{N_{g}}{\underset{g=1}{\sum }}\overset{N_{s}-1}{\underset{a=1}{\sum }}{|{\mathrm{c̃}}_{a}^{\left(g\right)}\left(0\right)|}^{2}\left\langle \Psi^{\left(g,a\right)}\left(t\right)\left|Â\right|\Psi^{\left(g,a\right)}\left(t\right)\right\rangle$$

We schematize the AXE in the absence of initial coherences with Figure 1(b, left), where for each geometry *g*, all states *a* = 1, 2, 3 are valid active states for the initial conditions without initial coherence. The AXE trajectories used in eq 15 only include the electronic coherences ${[c_{i}^{\left(g,a\right)}\left(t\right)]}^{*}c_{i^{\prime }}^{\left(g,a\right)}\left(t\right)$ arising at CoIns. Initial PGCs, however, are not accounted for, since the action of the pump pulse is included only through the population weight $w_{g,a}=N_{g}^{-1}|{\mathrm{c̃}}_{a}^{\left(g\right)}\left(0\right){|}^{2}$ for the respective trajectories initialized in the available electronic states. We will address this drawback in Section 2.5 by repropagating the PGCs along these precomputed AXE trajectories. We note for completeness that, if coherence contributions are disregarded, eq 15 can be recast in terms of the evolution of the active state only, in analogy to eq 3.

The AXE has the advantage that it fully decouples the effect of the pump pulse from the TSH computations and the calculation of the matrix elements of interest. This, however, comes at the expense of the number of trajectories that need to be propagated, i.e., one for each initial geometry and for each excited state (*g*,*a*). In order to reduce the number of trajectories, one can select a different ensemble of initial conditions *j* = (*g*,*a*) in which, for a given initial geometry *g*, not all excited states are retained, but only those states *a* that are more likely to be excited. This can be achieved by generating a stochastic sample of initial conditions, with a probability function $p_{a}^{\left(g\right)}$ determining whether a geometry *g* and an initial excited state *a* are kept in the sample. We refer to this approach as the stochastically Selected eXcited-state Ensemble (SXE) and the corresponding trajectories are referred to as the SXE trajectories.

As discussed in detail in Appendix A, $p_{a}^{\left(g\right)}$ can be defined in terms of the population $|{\mathrm{c̃}}_{a}^{\left(g\right)}\left(0\right){|}^{2}$ generated by the pump pulse. However, this would lead to an ensemble of initial conditions explicitly dependent upon the pump pulse used, having the same downsides already discussed in Section 2.2. To circumvent this issue, one can define the probability ${\mathrm{p̅}}_{a}^{\left(g\right)}$ as

16 $${\mathrm{p̅}}_{a}^{\left(g\right)}=\frac{f_{0a}^{\left(g\right)}}{\mathrm{N̅}}$$

proportional to the oscillator strength $f_{0a}^{\left(g\right)}$ between the electronic ground state S_0_ and the electronic excited state S_*a*_. This is effectively equivalent to disregarding the spectral properties of the pump pulse, thus assuming that the excited-state population $|{\mathrm{c̃}}_{a}^{\left(g\right)}\left(0\right){|}^{2}$ is proportional to $f_{0a}^{\left(g\right)}$ , as it is the case to first order in perturbation theory. The renormalization factor $\mathrm{N̅}\geq \underset{g,a}{\max}\left\{f_{0a}^{\left(g\right)}\right\}$ ensures that ${\mathrm{p̅}}_{a}^{\left(g\right)}$ is lower than 1 and can thus be used as a probability. This resulting new ensemble S̅ of initial conditions {(*g*,*a*)}, referred to as the SXE, is illustrated in Figure 1(c, left) in which for each geometry *g*, only one state *a* = 1 or 2 or 3 is the active state for the initial conditions. The resulting expectation value of *Â* can then be obtained as an average over all the *N̅*_t_ trajectories in the resulting ensemble S̅

17 $$\left\langle {Â}_{\mathrm{SXE}}\left(t\right)\right\rangle =\frac{\mathrm{\alpha ̅}}{{\mathrm{N̅}}_{t}}\underset{\left(g,a\right)\in \mathrm{S̅}}{\sum }\frac{{|{\mathrm{c̃}}_{a}^{\left(g\right)}\left(0\right)|}^{2}}{{\mathrm{p̅}}_{a}^{\left(g\right)}}\left\langle \Psi^{\left(g,a\right)}\left(t\right)\left|Â\right|\Psi^{\left(g,a\right)}\left(t\right)\right\rangle$$

where the weight $w_{g,a}=\mathrm{\alpha ̅}{\mathrm{N̅}}_{t}^{-1}|{\mathrm{c̃}}_{a}^{\left(g\right)}\left(0\right){|}^{2}/{\mathrm{p̅}}_{a}^{\left(g\right)}$ is included to properly account for the populations generated by the pump pulse used. *N̅*_t_ is the total number of trajectories in the sample, associated with *N*_g_ different geometries, and $\mathrm{\alpha ̅}=\underset{N_{g}\to \infty }{\lim}\left\{{\mathrm{N̅}}_{t}/N_{g}\right\}$ (see Appendix A for more details). In analogy to eq 15, also eq 17 does not contain contributions from the initial coherences generated by the pump pulse. We note again for completeness that, if coherence contributions are disregarded altogether, then eq 17 can be recast in terms of active state wave functions, as introduced with eq 3.

The advantage of eq 17 is that it relies on an ensemble generated independently of the pump pulse and the populations $|{\mathrm{c̃}}_{a}^{\left(g\right)}\left(0\right){|}^{2}$ , and that compared to eq 15, supposedly less trajectories need to be propagated. The possible downside is, however, that the ensemble generated via the oscillator strengths can differ quite significantly from the ensemble that would be generated based on the populations actually excited by the pump. In general, the prefactor $|{\mathrm{c̃}}_{a}^{\left(g\right)}\left(0\right){|}^{2}/{\mathrm{p̅}}_{a}^{\left(g\right)}$ in eq 17 is supposed to correct for this by properly including the effect of the pump pulse and undo the bias of the sampling based on the oscillator strengths. It is used similarly to the weighting procedure introduced in ref 70 where the same observable is evaluated from different probability distribution functions (without having to repeat the procedure for different samples). However, for a highly biased ensemble, i.e., an ensemble in which the pulse favors states with lower oscillator strengths, this correction might turn out problematic and lead to slow convergence with respect to the number of initial conditions. An extension to *manually* unbias the sampling procedure is proposed in Appendix B.

We finally stress that the expectation values within the AXE and the SXE trajectories, eq 15 and eq 17, respectively, converge to the same quantity for a sufficiently large number of initial conditions (*N*_g_ and *N̅*_t_, respectively). In summary, the expectation value ⟨*Â*(*t*)⟩ of any operator is thus accessible if its matrix representation in the adiabatic states can be obtained from quantum chemistry packages (see eq 15 and eq 17). Neglecting the contributions from coherences arising at CoIns, one can further reduce the number of matrix elements needed, focusing only on the properties of the active state *b* ^(*j*)^(*t*), in analogy to eq 3.

### 2.5 Postprocessing Trajectories in the Presence of Initial Coherences

Owing to the choice for the initial state in the two presented ensemble approaches (see, e.g., eq 14) and the form of the expectation values in eq 15 and eq 17, PGCs cannot be accounted for. We hereafter propose an extension to the AXE and the SXE approaches, eq 15 and eq 17, respectively, to account for the initial PGCs at negligible computational cost, postprocessing trajectories and matrix elements previously computed in the absence of PGCs. The proposed extension consists of two steps. In a first step, the initial conditions, defined either with AXE or the SXE, are used to precompute the nonadiabatic trajectories, propagating both electronic and nuclear degrees of freedom in the absence of initial coherences with $c_{i}^{\left(g,a\right)}\left(0\right)=\delta_{ia}$ . This yields the trajectories ***R*** ^(*g*,*a*)^(*t*), from which all necessary matrix elements associated with the desired observables can be computed. It corresponds to the case shown in Figure 1(b and c, left). In order to account for initial coherences in a postprocessing manner, we repropagate, in a second step, the electronic dynamics along the previously computed, *frozen* trajectories ***R*** ^(*g*,*a*)^(*t*), as described in Section 2.3, from the new initial coefficients $c_{i}^{\left(g,a\right)}\left(0\right)$ defined via eq 10. This is illustrated by the repropagation step in Figure 1(b and c, right) for the AXE and the SXE trajectories, reflecting the postprocessing of the electronic dynamics with the inclusion of the PGCs.

For the repropagated electron dynamics along the AXE trajectories, the expectation values are computed according to

18 $$\left\langle {Â}_{\mathrm{RP}‐\mathrm{AXE}}\left(t\right)\right\rangle =\frac{1}{N_{g}}\overset{N_{g}}{\underset{g=1}{\sum }}\overset{N_{s}-1}{\underset{a=1}{\sum }}{|{\mathrm{c̃}}_{a}^{\left(g\right)}\left(0\right)|}^{2}\left\langle \Phi^{\left(g,a\right)}\left(t\right)\left|Â\right|\Phi^{\left(g,a\right)}\left(t\right)\right\rangle$$

where the repropagated (RP) electronic wave function is now given by

19 $$\left|\Phi^{\left(g,a\right)}\left(t\right)\right\rangle =\overset{N_{s}-1}{\underset{i=0}{\sum }}C_{i}^{\left(g,a\right)}\left(t\right)\left|\psi_{i};R^{\left(g,a\right)}\left(t\right)\right\rangle$$

Note that the coefficients $C_{i}^{\left(g,a\right)}\left(t\right)$ are the newly repropagated electronic coefficients obtained via eq 12 by solving the TDSE along the frozen precomputed AXE trajectories ***R*** ^(*g*,*a*)^(*t*), starting from an initial coherent superposition, $C_{i}^{\left(g,a\right)}\left(0\right)$ = $c_{i}^{\left(g,a\right)}\left(0\right)$ (see also eq 10). They are used to compute the individual expectation values

⟨Φ(g,a)(t)|Â|Φ(g,a)(t)⟩=∑i,i′=0Ns−1[Ci(g,a)(t)]*Ci′(g,a)(t)×⟨ψi;R(g,a)(t)|Â|ψi′;R(g,a)(t)⟩ 20

Similarly, for the repropagated electron dynamics along the SXE trajectories, the expectation values are now given by

21 $$\left\langle {Â}_{\mathrm{RP}‐\mathrm{SXE}}\left(t\right)\right\rangle =\frac{\mathrm{\alpha ̅}}{{\mathrm{N̅}}_{t}}\overset{{\mathrm{N̅}}_{t}}{\underset{\left(g,a\right)\in \mathrm{S̅}}{\sum }}\frac{{|{\mathrm{c̃}}_{a}^{\left(g\right)}\left(0\right)|}^{2}}{{\mathrm{p̅}}_{a}^{\left(g\right)}}\left\langle \Phi^{\left(g,a\right)}\left(t\right)\left|Â\right|\Phi^{\left(g,a\right)}\left(t\right)\right\rangle$$

with individual expectation values computed based on the corresponding repropagated electronic coefficients $C_{i}^{\left(g,a\right)}\left(t\right)$ in analogy with eq 20. The total expectation values are thus computed without having to repeat calculations for the *frozen* trajectories ***R*** ^(*g*,*a*)^(*t*), nor for the corresponding, computationally expensive matrix elements ⟨ψ_*i*_; ***R*** ^(*g*,*a*)^(*t*)|*Â*|ψ_*i*′_; ***R*** ^(*g*,*a*)^(*t*)⟩, but only require the computationally inexpensive repropagation of the electronic coefficients $C_{i}^{\left(g,a\right)}\left(t\right)$ via eq 12, including the initial PGCs and electronic coefficients $C_{i}^{\left(g,a\right)}\left(0\right)=c_{i}^{\left(g,a\right)}\left(0\right)$ at the postprocessing stage.

In the following section, in order to numerically validate these repropagation approaches based on AXE and SXE trajectories, we compare results obtained by (i) including the initial PGCs right from the start in the propagation of both electronic and nuclear degrees of freedom, as discussed in Section 2.2 and (ii) including these PGCs only in the repropagation of the electronic degrees of freedom on previously computed trajectories and dynamics, as presented here. In the following, we refer to the repropagation of the electronic coefficients and their use to compute expectation values with eq 18 and eq 21 as repropagated PGCs along the AXE and SXE trajectories, for short, RP-AXE and RP-SXE, respectively.

We note that in all three FP, RP-AXE, and RP-SXE approaches accounting for the PGCs, the observable can be recast as

22 $$\left\langle Â\left(t\right)\right\rangle =\overset{N_{t}}{\underset{j=1}{\sum }}w_{j}A_{j}\left(t\right)$$

running over different nuclear ensembles with corresponding weights *w*_*j*_ and individual expectation values *A*_*j*_(*t*). The variables acting as *A*_*j*_(*t*) and *w*_*j*_ for the three approaches are summarized in Table 1.

**Table 1:** Definition of the Notations for the Three Presented Nuclear Ensemble Approaches, FP, RP-AXE, and RP-SXE, Accounting for Initial PGCs

Approach	Traj. index *j*	Sample size *N*_t_	*w*_*j*_	*A*_*j*_(*t*)
FP	*g*	*N*_g_	$N_{g}^{-1}\overset{N_{s}-1}{\underset{k=1}{\sum }}|{\mathrm{c̃}}_{k}^{\left(g\right)}\left(0\right){|}^{2}$	⟨Ψ^(*g*)^(*t*)|*Â*|Ψ^(*g*)^(*t*)⟩
RP-AXE	(*g*,*a*)	(*N*_s_ – 1)×*N*_g_	$N_{g}^{-1}|{\mathrm{c̃}}_{a}^{\left(g\right)}\left(0\right){|}^{2}$	⟨Φ^(*g*,*a*)^(*t*)|*Â*|Φ^(*g*,*a*)^(*t*)⟩
RP-SXE	(*g*,*a*) ∈S̅	*N̅*_t_	$\mathrm{\alpha ̅}{\mathrm{N̅}}_{t}^{-1}|{\mathrm{c̃}}_{a}^{\left(g\right)}\left(0\right){|}^{2}/{\mathrm{p̅}}_{a}^{\left(g\right)}$	⟨Φ^(*g*,*a*)^(*t*)|*Â*|Φ^(*g*,*a*)^(*t*)⟩

For the sake of visualization, instead of employing the expectation values in eq 11, eq 18 and eq 21, the results in Section 3 will be displayed employing the corresponding weighted averages given in eq 36, eq 39, and eq 43 for the PGCs included from the start (FP) and repropagated along AXE and SXE trajectories (RP-AXE, RP-SXE), respectively (see Appendix C). This allows for an easier comparison of the expectation values ⟨*Â*(*t*)⟩ with the individual expectation values *A*_*j*_(*t*) for the independent individual trajectories.

## 3 Results and Discussion

### 3.1 Model System Based on Glycine

We choose the neutral glycine I_p_ conformer and its electronic excited states as a flexible test case for illustrating the presented methodologies. The electronic structure of the first five singlet electronic states of the molecule is evaluated at the CASSCF level of theory with six active electrons in four valence orbitals, referred to as CAS (6,4)-SA5 in the following. More details regarding the electronic structure and the parameters for nonadiabatic dynamics are given in the Section 5. Both the Lewis structure of the molecule and the geometries associated with the harmonic approximation of the Wigner distribution are shown in Figure 2(a) and (b), respectively. The orientation of the molecule is chosen such that *z* is the direction normal to the symmetry plane of the molecule. For the chosen orientation, the transition dipole moments between the ground state S_0_ and the first two electronic excited states S_1_ and S_2_ are aligned along the *z* direction, while the transition dipole moment between S_0_ and the third electronic excited state S_3_ lies in the *x*–*y* plane. The fourth electronic excited state S_4_ is dark in all directions, as it cannot be excited from the ground state S_0_. The transition energies and dipoles of the first three electronic transitions at the minimum of the electronic ground state are given in Figure 2(c). The strength of the different transitions is reflected in the absorption spectrum depicted in Figure 2(d), here shown assuming a pulse with constant spectral intensity. The spectrum exhibits the largest peak for absorption from the ground state to the S_3_ state, reflecting the large transition dipole moment associated with this transition.

**Figure 2:**
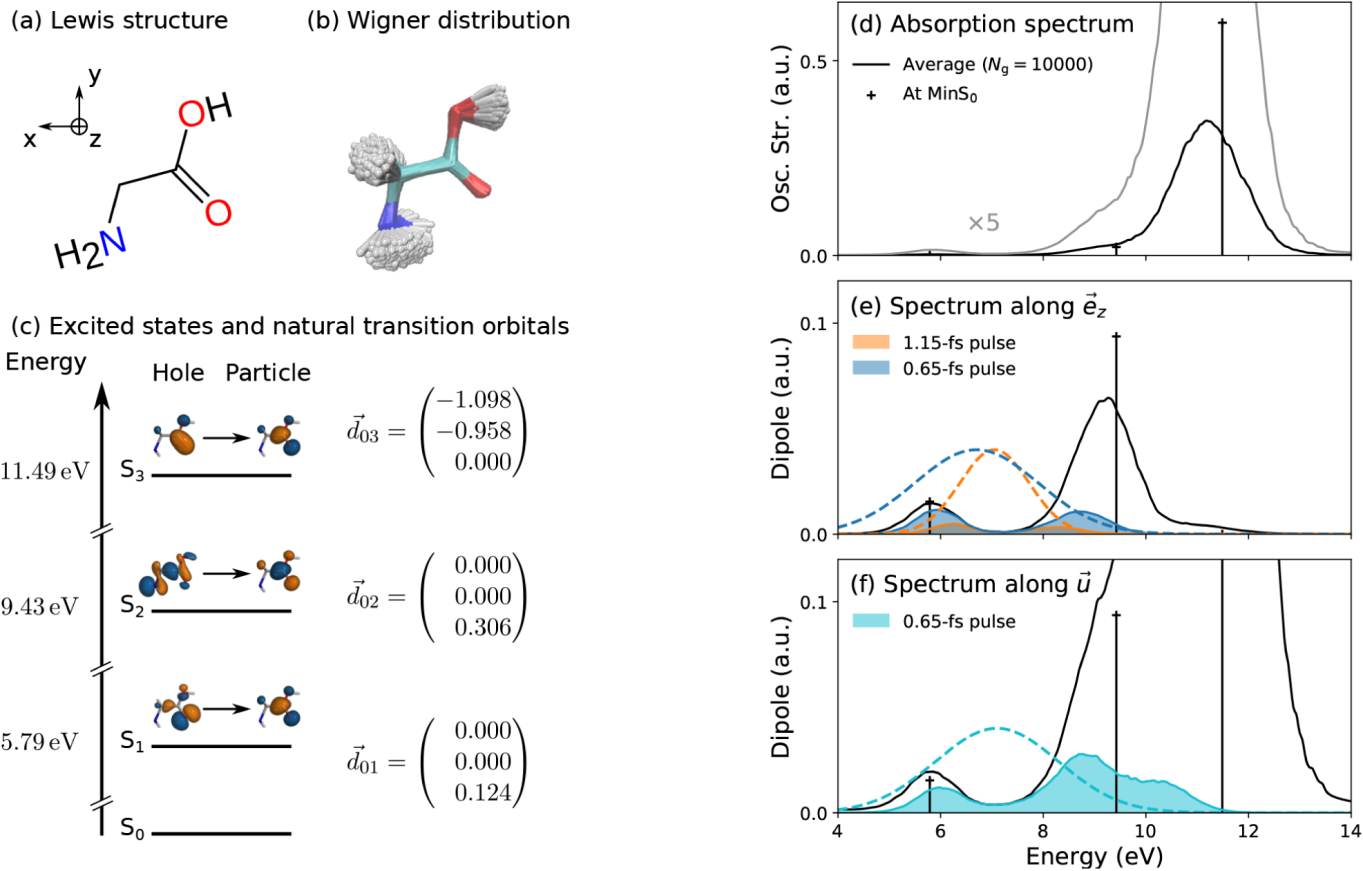
(a) Lewis structure of glycine and Cartesian axes. (b) Superposition of the *N*_*g*_ = 10,000 geometries of the Wigner distribution. (c) First three electronic excited states of glycine at the CAS­(6,4)-SA5 level of theory. The first hole and particle natural transition orbitals, the electronic transition dipole moments (in atomic units) and the transition energies (in eV) are given. (d) Steady-state absorption spectrum of glycine at the minimum of the electronic ground state (vertical lines) and as an average over the Wigner distribution (continuous black line) broadened with a Gaussian line shape (fwhm = 0.1 eV). The gray line represents the same spectrum multiplied by 5 to highlight the nonzero absorption of S_1_. (e, f) Absorption spectra in terms of dipole strengths and associated convolved spectra with different pulses along two field directions, *e⃗*_*z*_ and a more general *u⃗*, further described in Sections 3.2 and 3.4, respectively. The pulse spectra $|\overset{⃗}{Ẽ}\left(\omega \right){|}^{2}$ are displayed in dashed lines. The direction *e⃗*_*z*_ ensures that only the first two electronic excited states absorb, whereas the direction *u⃗* is chosen such that there is absorption to all three electronic excited states.

### 3.2 Realistic Pulse for Initial Pump-Generated Coherences

#### 3.2.1 Pump-Generated Electronic Coefficients

In a previous study, the dynamics induced by an exact superposition of S_1_ and S_2_ electronic excited states in glycine were investigated by artificially setting the corresponding amplitudes to $c_{1}=c_{2}=1/\sqrt{2}$ for all initial geometries.^67^ This situation corresponds to an ad hoc, ideal pump pulse exciting a perfect superposition of the two states. Here, instead of this idealized scenario, we explicitly consider the excitation by a broadband pulse, modeled by the Gaussian spectral envelope

23 $$\overset{⃗}{Ẽ}\left(\omega \right)={Ẽ}_{0}\;\exp\left(-\frac{{\left(\omega -\omega_{L}\right)}^{2}}{2\sigma^{2}}\right)\mathrm{p⃗}$$

where *Ẽ*_0_ is the strength of the pulse (set to *Ẽ*_0_ = 1 in the following), ω_L_ is the pulse central frequency, the width $\sigma =\Delta \omega /\left(2\sqrt{\ln2}\right)$ is related to the full width at half-maximum (fwhm) Δω of $|\overset{⃗}{Ẽ}\left(\omega \right){|}^{2}$ , and *p⃗* is the unitary polarization vector of the field. In the presence of such envelope, the initial amplitudes ${\mathrm{c̃}}_{i}^{\left(g\right)}\left(0\right)$ of the electronic states following pump excitation are then obtained from first-order perturbation theory as

$${\mathrm{c̃}}_{k}^{\left(g\right)}\left(0\right)=i{\mathrm{d⃗}}_{0k}\left(R^{\left(g\right)}\left(0\right)\right)\cdot \overset{⃗}{Ẽ}(\omega_{k0}\left(R^{\left(g\right)}\left(0\right)\right),,\forall k\neq 0$$ 24

where *d⃗*_0*k*_(***R*** ^(*g*)^(0)) and ω_*k*0_(***R*** ^(*g*)^(0)) are the corresponding dipole moments and transition energies between the ground state S_0_ and the excited state S_*k*_ at the initial geometry ***R*** ^(*g*)^(0). In the following, the electronic coefficients (see eq 24) are used as initial conditions (see eq 4 and eq 10) for PGCs included from the start and for the repropagated PGCs along the AXE and the SXE trajectories. In eq 24, the time *t* = 0 represents the end of the pulse *E⃗*(*t*), where *E⃗*(*t*) is the Fourier transform of $\overset{⃗}{Ẽ}\left(\omega \right)$ . Equation 24 thus lies on the assumption that the nuclei are frozen during the interaction between the molecule and the ultrashort pulse. This short-time scale interaction was recently studied, notably with the use of the promoted density approach, see ref 71 and shown to be indeed negligible for pulses as short as those employed in this work, see Section 3.2.2 and Figure 2(e, f).

We will now employ the computational approaches introduced in Section 2 to predict the evolution of the electronic populations $P_{i}^{\left(g\right)}\left(t\right)=|c_{i}^{\left(g\right)}\left(t\right){|}^{2}$ , the electronic coherences Re([ci(g)(t)]*ci′(g)(t)) , and the expectation value of the permanent molecular dipoles *d⃗* ^(^ *^g^* ^)^(t) following from the initial electronic coefficients in eq 24. Thereby we scrutinize the effectiveness of postprocessing PGCs along the AXE and SXE trajectories for reproducing the dynamics obtained by including PGCs from the start.

#### 3.2.2 Choice of the Initial Pulse

In order to investigate the influence of the pulse properties on the initial state amplitudes, and thus verify the possibility to generate a coherent superposition of electronic states with a realistic pump pulse, we first consider two pulses both aligned along the *z* direction in the molecular frame, *p⃗* = *e⃗*_*z*_, but with different laser central frequency ω_L_ and width Δω, i.e., {ω_L_ = 7.05 eV, Δω = 1.59 eV} and {ω_L_ = 6.70 eV, Δω = 2.83 eV}. In time domain, these correspond to pulse durations (fwhm of |*E⃗*(*t*)|^2^) of approximately 1.15 and 0.65 fs, respectively, which are now accessible or close to accessible in several laboratories.^5,6^ In the following, we refer to these two pulses as the 1.15 fs and the 0.65 fs pulses, respectively. The central frequency and width of the pulses are chosen to maximize the excitation of the first two excited states S_1_ and S_2_ and minimize the excitation of S_3_. The spectrum for the two pulses in Figure 2(e) shows that an approximately equivalent excitation of the two states is indeed possible. However, the spectra do not reflect any information about the initial coherence between the two states. To better characterize the initial coherences generated by the pulse, we show in Figure 3(a)–(d) the distributions of both population difference $P_{2}^{\left(g\right)}\left(0\right)-P_{1}^{\left(g\right)}\left(0\right)$ and coherence Re([c1(g)(0)]*c2(g)(0)) for the considered sample of geometries, comparing results for the 1.15 and 0.65 fs pulses, respectively.

**Figure 3:**
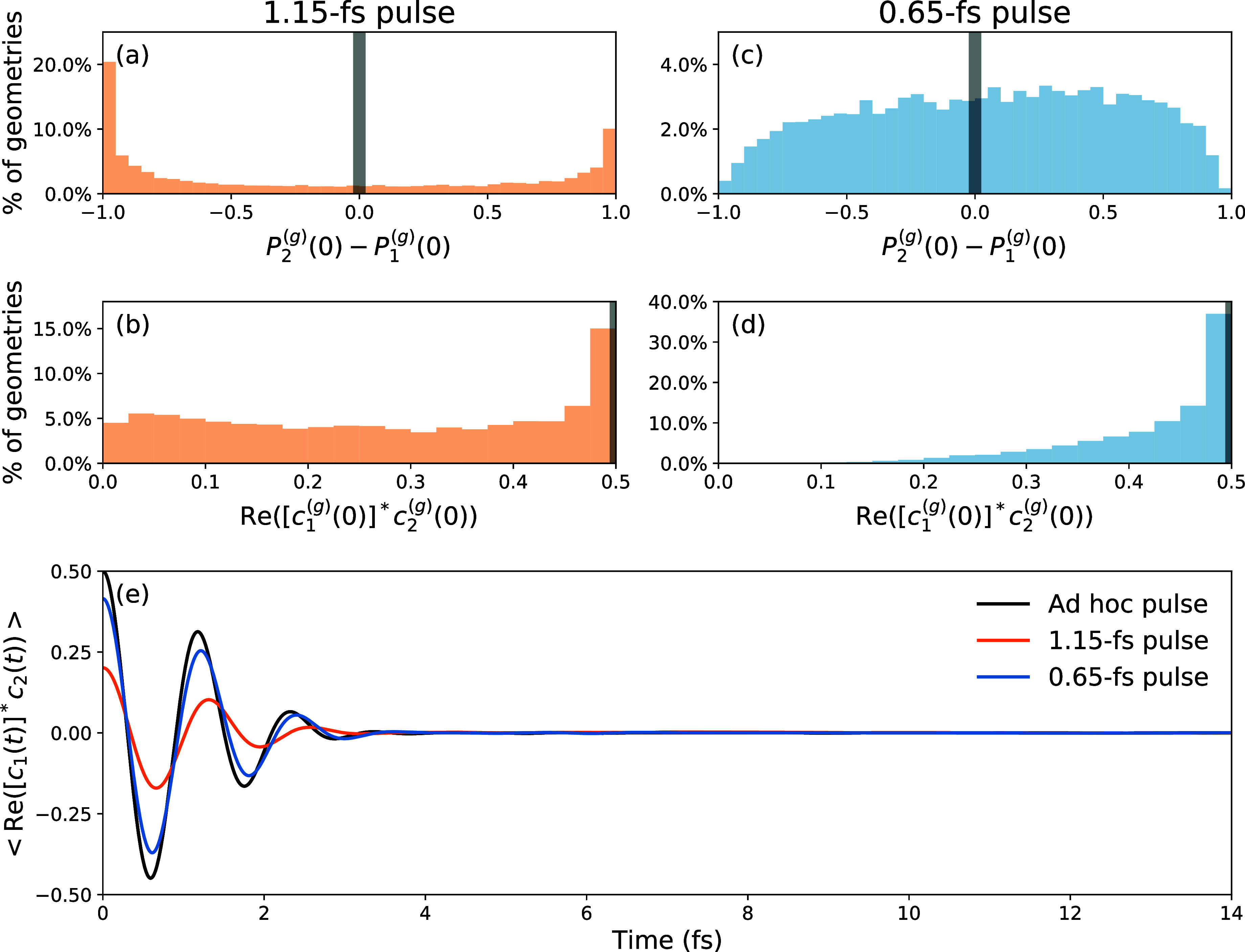
Panels (a, b): histograms of (a) the initial population difference and (b) the initial coherence, for the initial Wigner sample of geometries interacting with the 1.15 fs Gaussian pulse {ω_L_ = 7.05 eV, Δω = 1.59 eV}. Panels (c, d): same as in panels (a, b) for the initial Wigner sample of geometries interacting with the 0.65 fs Gaussian pulse {ω_L_ = 6.70 eV, Δω = 2.83 eV}. The gray broad lines correspond to the ideal but ad hoc pulse exciting both states equally, with maximum initial coherence. Panel (e): evolution of the coherence between states S_1_ and S_2_ with the PGCs included from the start with eq 36 for the ad hoc pulse, the 1.15 fs pulse, and the 0.65 fs pulse in black, orange, and blue, respectively.

For the 1.15 fs pulse, Figure 3(a) indicates that in most geometries, either S_1_ or S_2_ are highly populated. As a result, a maximum coherence of 0.5 is obtained only for about 15% of the sample, whereas the remaining 85% corresponds to coherence values between 0.025 and 0.475, see Figure 3(b). This is consistent with close-to-one populations either in S_1_ or S_2_. In contrast, for the 0.65 fs pulse, the populations are more evenly distributed between the two extremes, see Figure 3(c). Almost 40% of the initial sample is promoted to a coherence close to the maximum of Re­([*c*_1_(0)]^*^ *c*_2_(0)) ≃ 0.5, as displayed in Figure 3(d). The different coherent superpositions generated by the two pulses are reflected in the time evolution of the coherence, shown in Figure 3(e) for the two pulses. The results indicate that the decoherence occurs within 3 fs, encompassing three full oscillation periods with amplitudes depending on the strength of the initial PGCs. For completeness, we also depict the distributions of the individual $c_{i}^{\left(g\right)}\left(0\right)$ coefficients for the two pulses in Figure S1 of the Supporting Information, and check how the two situations are comparable to an ideal two-state scenario, approximately satisfying |*c*_1_|^2^ + |*c*_2_|^2^ = 1, see Figure S2 of the Supporting Information.

We note that the 0.65 fs pulse generates an initial coherence (Re­([*c*_1_(0)]^*^ *c*_2_(0)) = 0.41) close to the maximum possible coherence with the ad hoc pulse, while the 1.15 fs pulse, albeit more realistic, initializes the dynamics with an initial coherence of Re­([*c*_1_(0)]^*^ *c*_2_(0)) = 0.21. In the following, we will focus on the dynamics initialized by 0.65 fs pulse, as it yields the largest initial coherence. For most of the displayed figures for the 0.65 fs pulse, we provide equivalent ones for the ad hoc pulse and the 1.15 fs pulse in Figure S3 and Figure S4 of the Supporting Information, respectively.

### 3.3 Nonadiabatic Dynamics Induced by a 0.65 fs UV Pulse in a Two-State Case

#### 3.3.1 Fully Including the Pump-Generated Coherences

We show now how the initial coherent superposition generated by an ultrashort pulse affects the early coupled electron–nuclear dynamics. We will explicitly make use of the 0.65 fs pulse described above, which coherently excites two electronic states of glycine. The ensuing nonadiabatic dynamics of both electronic and nuclear degrees of freedom are computed here with TSH-PFM by taking into account the PGCs from the start, with nonzero $c_{1,2}^{\left(g\right)}\left(0\right)$ electronic coefficients given by eq 24. This corresponds to the situation described in Figure 1(a), with the expectation values computed according to eq 11. For visualization and interpretation purposes, in order to more easily compare the sample-averaged observables with the expectation values of the individual trajectories, the physical observables shown in this section and their evolution are computed according to the weighted average given by eq 36 (see also Appendix C).

The time evolutions of the S_1_ population, the S_1_–S_2_ electronic coherence, the molecular dipole strength, and its *x*, *y*, and *z* components are shown in Figure 4(a)–(d), respectively. Figure 4(a) and (b) highlight the decoherence between the two electronic states. The S_1_ population stays mostly constant over the first few femtoseconds, and the electronic coherence decays from its high initial value of 0.41 to almost zero in 4 fs, exhibiting three full oscillation periods with non-negligible amplitudes. The fast decay of the initial coherence is due to (i) the decoherence of the electronic wave functions along individual trajectories, here described by means of the PFM decoherence correction, and (ii) the dephasing of the electronic wave packets on the individual trajectories, leading to cancellation effects in the ensemble average, as usually highlighted in the literature.^60^

**Figure 4:**
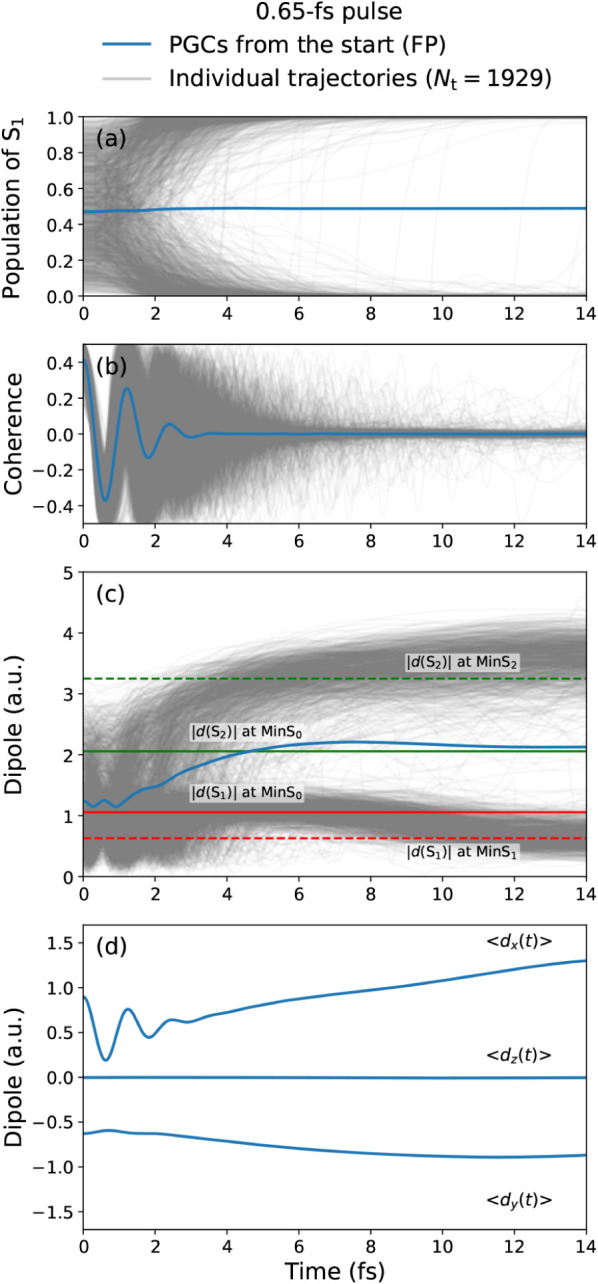
Nonadiabatic dynamics obtained with the FP approach, following the excitation of an initial coherent superposition with a 0.65 fs pulse in the presented glycine model. For all panels except (d), both the individual quantities for each trajectory (gray lines) and the total expectation values according to eq 36 (blue line) are given. Panel (a): Population *P*_1_(*t*) = |*c*_1_(*t*)|^2^ of the first electronic excited state S_1_. Panel (b): Coherence Re­([*c*_1_(*t*)]^*^ *c*_2_(*t*)). Panel (c): Molecular dipole strength, with reference horizontal lines showing the permanent dipoles of S_1_ (red) and S_2_ (green) at optimized geometries of the S_0_ state (solid) and of the S_1_, S_2_ states (dashed). Panel (d): Molecular dipole components along *x*, *y*, and *z* directions.

For more physical insight into the pump-induced dynamics, we present the evolution of the molecular dipoles (strengths and individual components) in Figure 4(c) and (d), which highlight their dependence on population and coherence contributions, as expected from eq 6 to eq 8. The ensemble of trajectories splits into two groups, propagating along the S_1_ or the S_2_ state in the late dynamics, respectively. During the early dynamics, the average dipole exhibits notable oscillations due to the contribution from the initial coherences, surviving for the first few femtoseconds. This is particularly well illustrated by the *x* component of the molecular dipoles, with oscillations of about 0.8 a.u. during the first femtoseconds after excitation, following the corresponding oscillations of the initial coherence.

Our results predict a strong effect of the initial coherent dynamics on the molecular dipoles. Looking back again at the form of the expectation value obtained for individual trajectories, eq 6 and eq 8, this strong influence can be ascribed to the large excited-state transition dipole moment *d*_12,*x*_. This observation can be relevant to design systems and experiments aiming at controlling molecular dynamics in neutral molecules by generating broadband electronic wave packets with ultrashort UV pulses.

#### 3.3.2 Postprocessing the Pump-Generated Coherences

As highlighted in Figures 3 and 4, initial electronic coherences can have an important effect on the subsequent dynamics, which can be accessed and monitored by time-resolved signals such as, e.g., time-resolved photoelectron spectroscopy or attosecond transient absorption spectroscopy. However, computing the evolution of the physical observables involved in these time-resolved experiments can be computationally expensive. Employing the TSH-PFM approach with PGCs included from the start, as in Figure 4, renders such computationally expensive signals dependent upon the pump-pulse characteristics, as it incorporates from the start the dependence of the physical observables on the PGCs. As such, it requires completely new calculations whenever the properties of the pump pulse are varied. To avoid this problem, in Section 2.5, we have proposed a postprocessing scheme to include the effect of the initial PGCs by repropagating the electronic coefficients along precomputed AXE and SXE trajectories. In the following, we compare the results obtained with these approaches to those obtained by including the PGCs from the start.

As mentioned in Section 3.3, for visualization purposes we display normalized individual trajectories and the corresponding weighted average for the expectation value of the different observables (see eq 39 and eq 43 in Appendix C). The left column of Figure 5 presents results obtained by repropagating the PGCs along the AXE trajectories, with the expectation values computed according to eq 39. These results are compared with the dynamics obtained by including the PGCs from the start with the FP approach (see eq 36). The agreement for populations, coherences and dipole components is excellent for the early dynamics, and it is slightly worse at longer times, after 5 fs. The oscillations in both the coherence and in the *x* component of the molecular dipole components are almost perfectly reproduced. A similarly good agreement is also displayed in the right column of Figure 5 when repropagating the PGCs along the SXE trajectories, with expectation values computed according to eq 43.

**Figure 5:**
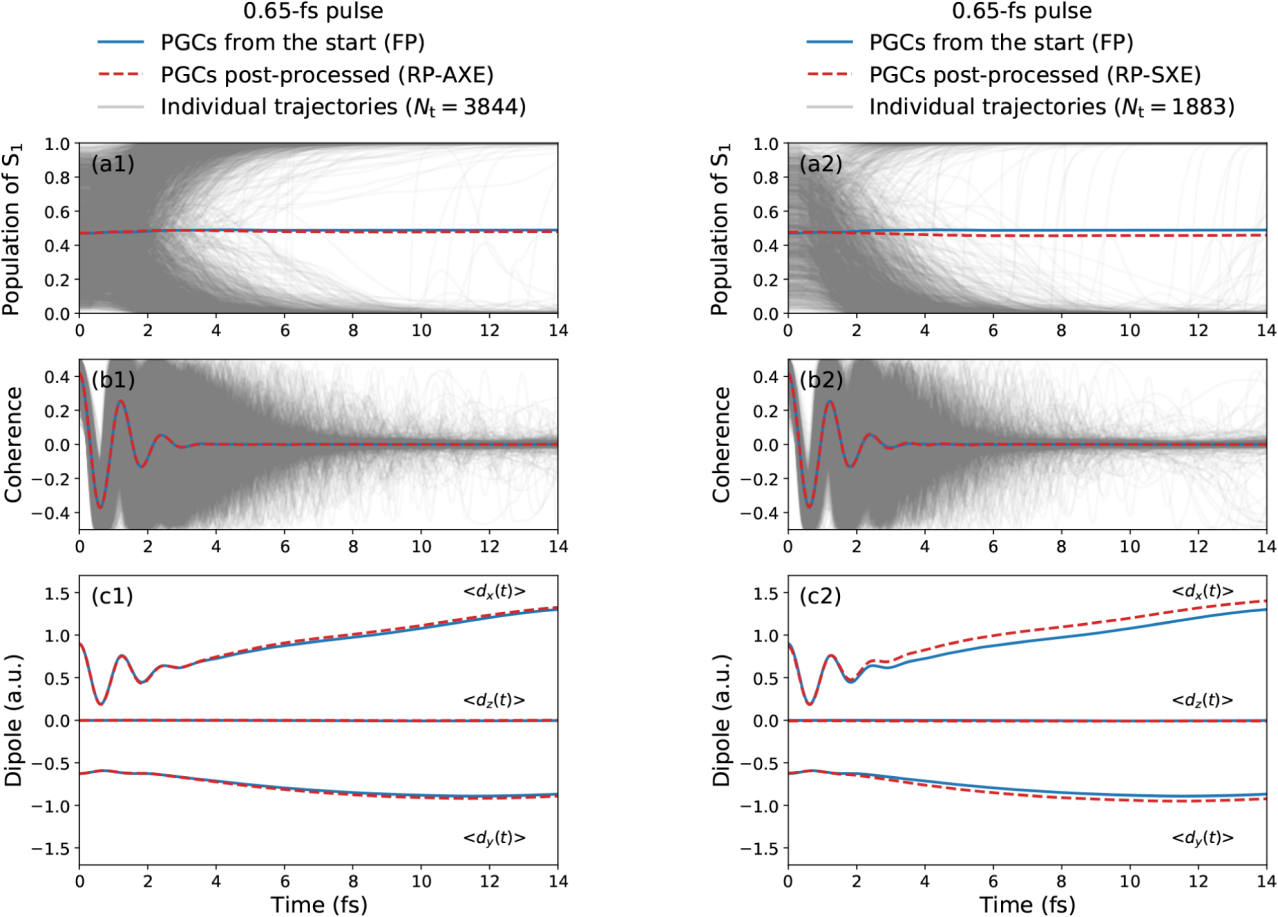
Quantities in panels (a1, a2), (b1, b2), and (c1, c2) are the same as in Figure 4(a), (b), and (d), respectively. The observables obtained by postprocessing the PGCs (dashed red lines), with repropagated electronic coefficients along AXE and SXE trajectories (gray lines), are compared to the observables obtained by including the PGCs from the start (solid blue lines). The left and right columns correspond to the repropagation of the PGCs along the AXE and the SXE trajectories, respectively (see eq 39 and eq 43 in Appendix C).

More apparent differences between results from the repropagation along the AXE and SXE procedures can be seen in the evolution of the populations for individual trajectories, see Figure 5(a1, a2). For the repropagated PGCs along the AXE trajectories, two equivalent groups of trajectories can be identified in Figure 5(a1), with populations moving toward the S_1_ or S_2_ state, respectively. In contrast, Figure 5(a2) for the repropagated SXE approach exhibits two inequivalently dense groups of trajectories, with the ones moving toward the S_2_ state being significantly more represented. This is due to the properties of the underlying ensembles, AXE or SXE, which determine the evolution of the active state *b* ^(*g*,*a*)^(*t*) along the corresponding precomputed trajectories. In the AXE, both excited states, S_1_ and S_2_, are equally represented in the sample of initial conditions. This leads to an ensemble of precomputed AXE trajectories in which, during the first 15 fs, S_1_ and S_2_ are equally represented as active states. Due to the decoherence correction employed here, when repropagating the electronic coefficients along these AXE trajectories starting from PGCs, the population $|C_{i}^{\left(g,a\right)}\left(t\right){|}^{2}$ of the inactive state gradually moves toward the one of the active state *b* ^(*g*,*a*)^(*t*). Each RP-AXE trajectory in Figure 5(a1) starts from a coherent superposition state in which the two excited states are equally populated, but the populations then quickly move to a single state, i.e., the active state for that specific trajectory, within the first 15 fs. The fact that S_1_ and S_2_ are equally represented as active states in AXE results in the two equally dense groups of trajectories in Figure 5(a1). Conversely, for the SXE [see Figure 5(a2)–(c2)], not all initial conditions are equally represented in the sample, as for a given geometry *g*, a corresponding trajectory (*g*,*a*) is retained in the sample with a probability proportional to the oscillator strength $f_{0a}^{\left(g\right)}$ of the considered state *a*. Since in general $f_{01}^{\left(g\right)}<f_{02}^{\left(g\right)}$ , the resulting sample is more likely to contain trajectories initiated in the active state S_2_. This leads to the two unequally dense groups of trajectories in Figure 5(a2): all the RP-SXE trajectories start from a superposition of almost equally populated states, but for a significant proportion of these trajectories, the populations are transferred completely to S_2_ within the first 15 fs. Nevertheless, the average populations resulting from the RP-SXE trajectories are in good agreement with both the FP and the RP-AXE ones. This is due to the sampling prefactor $|{\mathrm{c̃}}_{a}^{\left(g\right)}\left(0\right){|}^{2}/{\mathrm{p̅}}_{a}^{\left(g\right)}$ applied to the individual trajectories in eq 21 and eq 43, designed to account for this bias in the initial SXE. The same effect is even more noticeable when considering the ad hoc pulse with maximum initial coherences, see Figure S3 of the Supporting Information, using the same ideal superposition for all initial conditions. In this case, two equally or unequally dense groups of trajectories can be similarly identified, for the AXE and the SXE, respectively.

This same bias in the initial SXE also affects the convergence properties of the RP-SXE trajectories, as will be discussed in more detail in Section 3.3.3. For instance, in the two-state case shown in Figure 5(a2)–(c2), out of 1887 trajectories, only 146 were initialized in S_1_, with a clear bias toward S_2_. The weights $|{\mathrm{c̃}}_{a}^{\left(g\right)}\left(0\right){|}^{2}/{\mathrm{p̅}}_{a}^{\left(g\right)}$ are crucial to satisfy internal consistency with respect to the initial pump-generated populations $|{\mathrm{c̃}}_{a}^{\left(g\right)}\left(0\right){|}^{2}$ , on which the SXE is by construction independent. This same prefactor, however, gives a large weight to a specific group of initially under-represented trajectories, in this particular case, those initialized in the S_1_ state. This renders the overall convergence properties of the RP-SXE approach dependent on the convergence of a subset of trajectories. This is further illustrated in Figure S5 of the Supporting Information, where the weights of the trajectories initialized in the different possible initial active states are exhibited.

We finally note that, herein, we employed the PFM decoherence correction scheme for both the precomputed trajectories and the repropagation of the electronic coefficients along the frozen trajectories. In Figure S6 and S7 of the Supporting Information, we show similar results obtained by repropagating the electronic coefficients with the PFM decoherence correction but using trajectories precomputed with the TSH-EDC. Our results show that although TSH-EDC damps too quickly the initial coherences if PGCs are included from the start (FP approach), repropagating them on precomputed TSH-EDC trajectories with the PFM correction yields back the correct average behavior for the initial coherence and its decay. This indicates that in these conditions for glycine, the repropagation of the PGCs can be performed along precomputed trajectories that were not originally propagated with TSH-PFM, allowing for an even more flexible inclusion of the PGCs.

#### 3.3.3 Convergence of Repropagated Coherences

The RP-AXE and RP-SXE approaches feature different convergence properties with respect to the number of trajectories required. We highlight this by comparing the time evolution of the coherence with increasing number of initial conditions, shown in Figure 6(a1, b1) and (a2, b2) for the repropagation of the PGCs along the AXE and the SXE trajectories, respectively. From Figure 6(a1, a2), we see that for large coherence, within the very early dynamics, both methods rapidly reach convergence. The convergence of the late decoherence, on the other hand, happens to be significantly slower with the SXE trajectories, as can be seen in the inset of Figure 6(a2). We further exemplify this slow convergence by showing the coherence at specific times with decreasing amplitudes, Figure 6(b1) and (b2).

**Figure 6:**
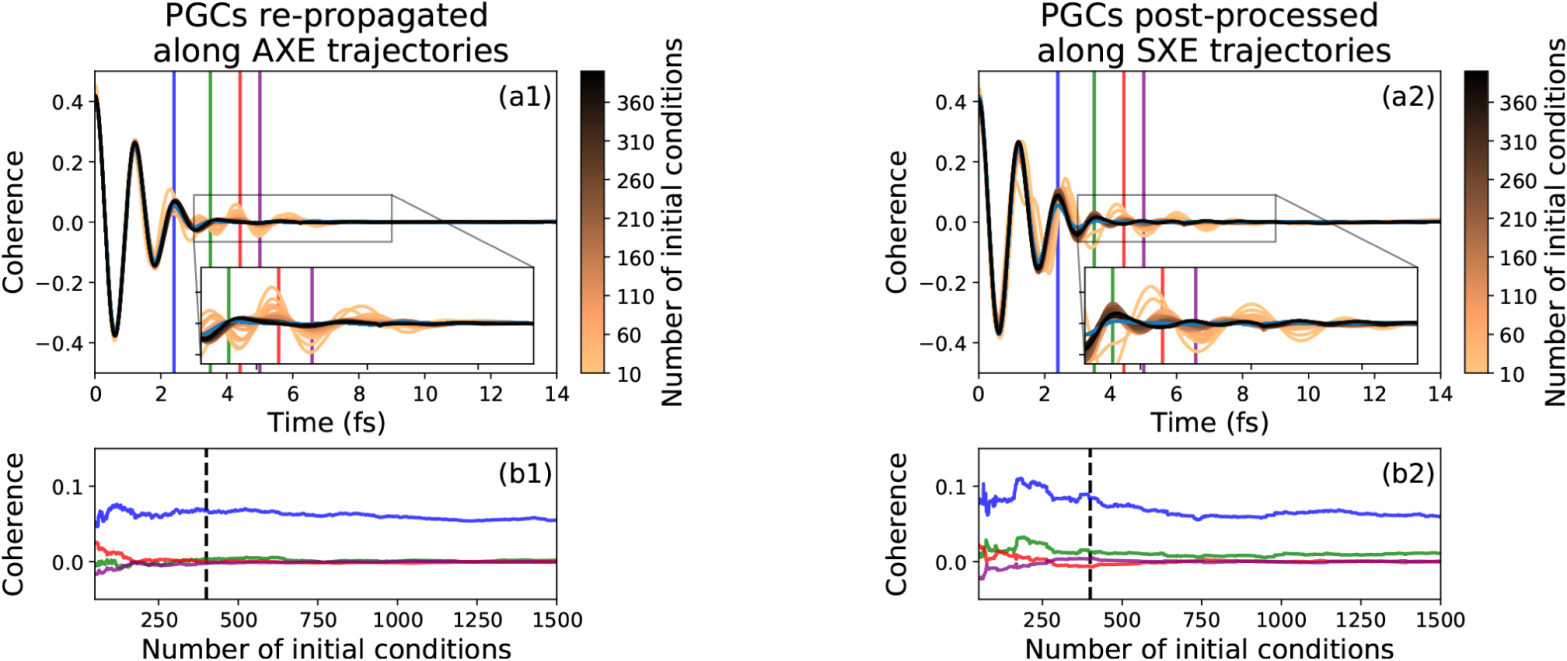
Convergence properties for the repropagation of the electronic coefficients $C_{i}^{\left(g,a\right)}\left(t\right)$ by using the AXE and the SXE trajectories, left and right, respectively. Panels (a1, a2): expectation value of Re([C1(g,a)(t)]*C2(g,a)(t)) where the gradient from orange to black indicates the number of propagated initial conditions retained. Panels (b1, b2): expectation value of Re([C1(g,a)(t)]*C2(g,a)(t)) at specific times as indicated by the vertical lines with the same colors in panels (a1, a2), as a function of the number of propagated initial conditions retained. For both the AXE and the SXE, the number of initial conditions runs over the geometry index *g*.

Overall, we can infer that the early decay (up to 3 fs) of the coherence quickly converges with the number of trajectories. The convergence becomes slower at later times when the averaged coherence approaches zero, although the individual trajectories still carry a significant coherence, see Figure 6(b1) and (b2). As mentioned in Section 3.3.2, we attribute this slower convergence of the RP-SXE approach to the fact that, to compensate for the difference between the pulse-independent oscillator strengths $f_{0a}^{\left(g\right)}$ and pulse-dependent initial populations $|{\mathrm{c̃}}_{a}^{\left(g\right)}\left(0\right){|}^{2}$ , large weights are assigned to trajectories (*g*,*a*) for which the initial active state *a* is significantly excited by the pulse despite having a relatively small oscillator strength. As a result, the convergence ultimately depends on the ensemble of trajectories started in the least favorable initial active state—in the present case, the trajectories whose initial active potential is S_1_. This is illustrated in Figure S5 of the Supporting Information, where we show the weights associated with trajectories started in different excited states. It is possible to render the convergence of the method less dependent on the oscillator strengths of the individual states by unbiasing the sample, i.e., by sampling different energy regions independently (see Appendix B), or directly using an unbiased sample such as in the AXE.

We note that the AXE seems to perform reasonably better than the SXE, although its computational cost is practically twice that of the SXE, as two (*N*_s_ – 1 in general) trajectories must be propagated for each geometry. However, the comparatively better performance of the AXE is also due to the fact that here, in order to test the capabilities of the RP-AXE and RP-SXE methodologies, we considered a particularly difficult scenario in which the pulse equivalently excites two states with significantly different oscillator strengths. In this case, AXE is by construction more suitable than SXE, since the latter introduces a significant bias via the selection criteria of the initial conditions in the sample of trajectories. However, for similarly bright states, one would expect AXE and SXE to have similar convergence properties. As such, the choice of the ensemble approach procedure for a given study must take into account how the spectral properties of the pump pulse differ from the steady-state absorption spectrum. Section 3.4 and Appendix B discuss how some of the limitations of the SXE can be circumvented.

### 3.4 Application to a Three-State Case

We have shown that postprocessing the PGCs by repropagating the electronic coefficients along the AXE and the SXE trajectories was efficient in a two-state case. However, this case is somehow limited to almost no population transfer and no particular nonadiabatic dynamics, which have been identified as ideal conditions for being able to repropagate electronic coefficients and postprocess PGCs according to eq 12. In this section, we further study the effectiveness of postprocessing the PGCs in a more complex scenario, allowing for the excitation of more than two states in glycine. For this purpose, we now consider the 0.65 fs pulse introduced in Section 3.2 aligned along the direction *p⃗* = *u⃗* = (−1.063, 0.932, 1.000)^T^, but slightly shifted in central frequency with respect to the previously defined 0.65 fs pulse, with {ω_L_ = 7.10 eV, Δω = 2.83 eV}. Aligning the polarization with such a vector *u⃗* with components in the three directions of the molecular frame allows the glycine molecules to absorb via all three excited states S_1_, S_2_, and S_3_. The absorption spectrum of glycine resulting from this pulse is shown in Figure 2(f). This corresponds to a situation where the first three excited states are populated, slightly in favor of S_2_. The initial populations are approximately *P*_*i*_(0) ≃ 0.2, 0.5, and 0.3 for the three states *i* = 1, 2, and 3, respectively.

The resulting population dynamics along with the coherences are shown in Figure 7. The solid cyan line displays results in which the initial PGCs are included from the start (see eq 39); whereas the dashed red lines are calculated by repropagating the PGCs along the AXE trajectories (a1–f1 panels) and along the SXE trajectories (a2–f2 panels) by using eq 39 and eq 45, respectively.

**Figure 7:**
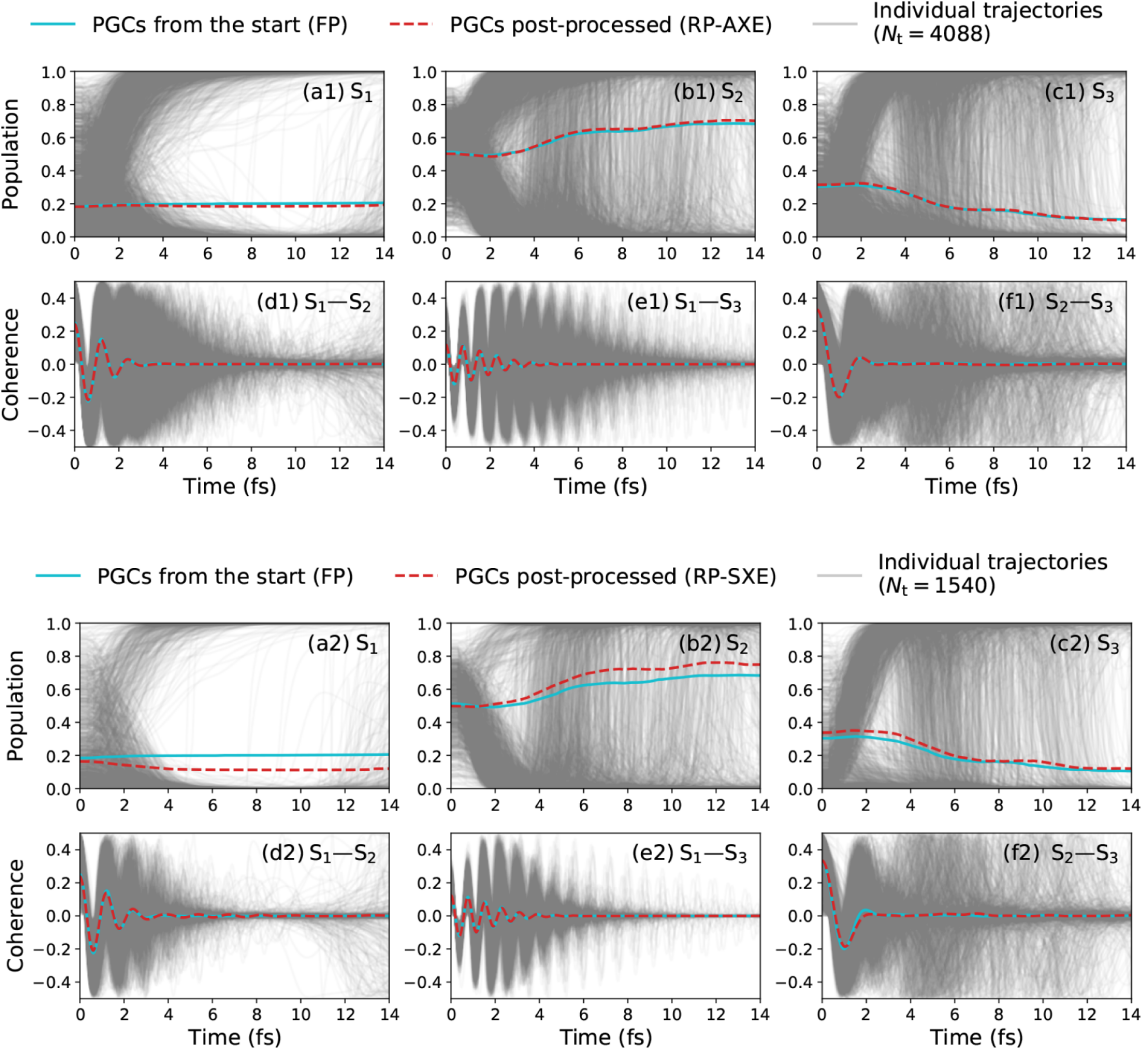
Nonadiabatic dynamics following the excitation of an initial coherent superposition with a 0.65 fs pulse in the presented glycine model. The observables obtained by postprocessing the PGCs (dashed red lines) with repropagated electronic coefficients along the AXE and SXE trajectories (gray lines) are compared to the observables obtained by including the PGCs from the start (solid blue lines). Panels (a, b, and c): Populations of states S_1_, S_2_, and S_3_, respectively. Panels (d, e, and f): Coherences between pairs of states S_1_–S_2_, S_1_–S_3_, and S_2_–S_3_, respectively. The top and bottom sets of six panels correspond to the repropagation of the PGCs along the AXE and SXE trajectories, respectively (see eq 39 and eq 45 in Appendix C).

Focusing first on the reference calculation (cyan solid lines, identically shown in (a1–f1) and (a2–f2) panels), the electronic dynamics now exhibit a population transfer from the S_3_ state to the S_2_ one, occurring in the first 8 fs. There is almost no transfer from or to the state S_1_. The pair coherences Re­([*C*_*i*_(*t*)]^*^ *C*_*i*′_(*t*)) are similarly non-negligible and oscillate for the first 4 fs, with oscillation frequencies related to the energy difference between the pair (*i*,*i*′) of states involved.

The repropagated PGCs along the AXE trajectories correctly reproduce both the population and early coherence dynamics, see Figure 7, top panels. Repropagating electron dynamics along the SXE trajectories (Figure 7, bottom panels) also leads to a correct description of coherences, although small differences with the results from including PGCs from the start can be seen in the population dynamics. We assume that this difference is due to the fact that the state S_1_ is not favored in the initial SXE due to its relatively smaller oscillator strength. For completeness, the dipole strength and components are given in Figure S8 of the Supporting Information. The agreement between PGCs included from the start and the repropagated PGCs along the AXE and SXE trajectories for the dipoles is consistent with the agreement already identified for populations and coherences.

In contrast to the two-state case of the previous sections, the three-state dynamics shown in Figure 7 feature population transfers and nonadiabatic dynamics, as evinced from the hops taking place in the individual trajectories, see, e.g., the frequent S_2_–S_3_ population exchange indicating hops, observed in panels (b1, c1) and (b2, c2). The overall agreement and good results obtained from the repropagated electronic dynamics thus corroborate the effectiveness of the RP-AXE and RP-SXE approaches also in the presence of population transfer. We note, however, that the majority of the active-potential hops between S_3_ and S_2_ occur after most of the initial PGCs have been damped, as can be seen from the S_2_ and S_3_ populations and S_2_–S_3_ coherence after 3 fs in Figure 7 (b1/b2, c1/c2, and f1/f2), respectively. The small number of hops occurring in this initial time window thus renders the repropagation approach of eq 12 a good approximation for predicting the evolution of the electronic coefficients $C_{i}^{\left(j\right)}\left(t\right)$ in the presence of PGCs. Under these conditions, it can then be expected that also the subsequent dynamics, once the PGCs are completely decayed, do not depend on the propagation (FP or RP) approach employed for the electronic dynamics. We further analyze the hopping times in Figure S9 of the Supporting Information by comparing how the occurrence of hops in the full-propagation trajectories and in the AXE trajectories are distributed in time. We observe that the hops take place at similar times in the full-propagation approach and in the AXE trajectories, and follow in both cases the decay of the coherence due to dephasing. Indeed, more than 90% of the hops take place after 3 fs, when the S_1_–S_2_ and S_2_–S_3_ coherences have already almost completely decayed, although the S_1_–S_3_ coherence keeps oscillating until about 4 fs. Our analysis for the three-state case confirms the applicability of the repropagation approach for trajectories in which a rare small number of hops take place in the presence of a PGC, which is a relatively common scenario when the molecular wave packet is generated far from conical intersections. However, for molecular wave packets generated at or close to a conical intersection, when a significant number of hops are expected to take place before the decay of the PGCs, further tests would be required to verify the applicability of the repropagation approach.

Before concluding, we note for completeness that the SXE was implemented here by using two separate energy regions, before and after 8.30 eV, described in more details in Appendix B. If a single sampling energy interval was used, the proportion of selected states would be approximately 1:10:200 for states S_1_, S_2_, and S_3_, respectively, consistent with the order of magnitudes of their oscillator strengths on which the SXE is based. For a total of *N* = 2000 initial conditions, only *N*_1_ = 10 initial conditions would be initialized in the potential of S_1_. However, since the initial pulse promotes 20% of population in S_1_, the weights $|c_{1}^{\left(g\right)}\left(0\right){|}^{2}/{\mathrm{p̅}}_{1}^{\left(g\right)}$ assigned to these *N*_1_ trajectories would be orders of magnitude larger than for the trajectories started in the other states. The convergence would be particularly slow, mostly dependent on the number of trajectories started in the S_1_ state (also previously discussed in Sections 3.3.2 and Section 3.3.3). Here, we manage to reduce the effect of these weights and thereby accelerate the convergence by using different sampling energy intervals for the S_1_ and S_3_ states, and correspondingly different N̅ for the two energy regions (see Appendix B).

## 4 Conclusions

We have presented two postprocessing approaches of the coupled electron–nuclear dynamics arising from a coherent superposition of electronic states, generated by realistic UV–vis broadband (ultrashort) pulses, based on two nuclear ensemble approaches, the AXE and SXE. The two approaches employ precomputed standard TSH trajectories independent of the pulse characteristics, thus avoiding recalculating the dynamics and the corresponding electronic structure information (energy gradients, matrix elements associated with observables, etc.) for each given pulse. The accuracy of the postprocessed results has been demonstrated for the case of aligned glycine molecules coherently excited to two and three electronic states by linearly polarized ultrashort UV pulses, by comparing the time evolution of state populations, coherences, and molecular dipoles with those obtained by incorporating the PGCs from the start of the dynamics. In all cases, the agreement between the latter and the former approaches has been found to be excellent. When repropagating the PGCs along the SXE trajectories, the agreement is slightly worse, yet with the advantage that it can allow one to work with a considerably smaller ensemble of trajectories. The strategy was tested for a two- and three-state scenario: in both cases, population transfer and corresponding active-potential hops were either very small or followed the decay of the initial PGCs, rendering the repropagation of the TDSE on precomputed nuclear trajectories a good approach for computing the electronic coefficients in the presence of initial PGCs. Yet, the repropagation strategy remains to be tested in cases where important population transfers, hence hops, take place while the initial coherences are still non-negligible.

Our developments pave the way for more robust and affordable trajectory-based ab initio simulations of pump–probe experiments, decoupling them from the dependence on the pump pulse characteristics, thus allowing for systematic calculations for the prediction and control of pump-generated nonadiabatic dynamics at a computational cost slightly higher than that required for a single pump pulse. The postprocessing approaches, applied here to coherent superpositions of electronic excited states in neutral molecules, can equally be applied to the study of initial electronic coherences in molecular cations. Future work on more complex molecules and more challenging scenarios of coupled electron–nuclear dynamics as, e.g., photoinduced chemical reactions and charge or energy intra- and intermolecular transfers, would be the next step to gauge the accuracy of this methodology in a broader context.

## 5 Computational Details

We briefly outline the computational details associated with both electronic structure theory and nonadiabatic dynamics.

### 5.1 Electronic Structure Calculations

All electronic structure calculations are performed using the openMolcas package.^72^ The geometry of the minimum of the electronic ground state S_0_ is optimized at the second-order many-body perturbation theory (MBPT2) and normal-mode frequencies are evaluated for this geometry. The cc-pVDZ basis set is used consistently for ground-state and excited-state calculations. The excited-state properties (energies, analytical gradients, and electronic transition dipole moments) are evaluated using the complete active space self-consistent field method (CASSCF) with the state-average (SA) formalism.^73,74^ The CAS consists of six electrons in one π orbital, two σ orbitals, and one π* orbital, see Figure S10 of the Supporting Information for the first five electronic states. It is referred to as CAS­(6,4)-SA5. The geometries of the minimum of the electronic excited states are optimized at the same CASSCF level of theory.

### 5.2 Trajectory-Based Nonadiabatic Dynamics

For the TSH calculations, a local modification of the SHARC package has been used.^75^ The Wigner distribution is sampled using the Fourier grid Hamiltonian method, generating 10000 geometries from the MBPT2-optimized minimum of S_0_ and the associated normal modes of vibration.^76^ Unless otherwise specified, the decoherence scheme is the projected forces and momenta approach with momentum injection, called TSH-PFM here and TSH-PFMi in ref 67. The decoherence parameter ω_PFM_ = 4.825 × 10^–3^ *E*_h_ is obtained as the geometric mean over the 24 normal-mode frequencies of glycine evaluated at the same geometry. The population threshold for the inactive-potential momentum propagation is η = 1 × 10^–4^. For an exhaustive description of the TSH-PFM algorithm, the reader is referred to ref 67.

The nuclear displacements are evaluated every time step Δ*t* = 0.3 fs following a velocity-Verlet algorithm, using the analytical gradients for the considered active state at time *t* from openMolcas, up to 14.1 fs. The electronic coefficients are propagated in a local diabatic basis, which is constructed such as to maximize the overlap between consecutive nuclear time steps for each electronic state, with 51 electronic substeps from one nuclear step to the other.^67,77^ The transition probabilities between adiabatic states are evaluated following the density flux formalism as originally proposed in ref 78.

In general, an ensemble of 2000 geometries is used. For the FP approach and for the SXE, it consists of 2000 initials conditions, each one characterized by an initial geometry *g* and an initial active state *a*. For the AXE, all excited states can be considered as active for each of the 2000 geometries, leading to a larger number of TSH initial conditions and trajectories. The choice of the active states in this situation is discussed in more details in Appendix D. In all three cases, the trajectories for which the energy is not conserved at all times within a tolerance threshold of Δ*E* = 0.5 eV are discarded. As a consequence, the final ensemble of trajectories needs to be *balanced* with respect to the number of discarded trajectories initialized in the most numerically unstable active state *a*. The balancing procedure is detailed in ref 67. For PGCs included from the start and for the SXE, the balancing procedure simply consists in further discarding trajectories *j* started in the other, more numerically stable initial states *a*′ ≠ *a*, in such a way that the initial ratios before and after checking the validity of the trajectories is conserved. For the AXE, by construction, if the trajectory initialized with (*g*,*a*) is discarded, then all the trajectories started in *g* are discarded, so that the geometry itself is fully discarded. The number of initially run, valid, and balanced trajectories is detailed in Table 2. For all three methodologies, around 5% and 25% of the trajectories are excluded in the two-state case and three-state case, respectively. This number is higher in the three-state case because SA-5 CAS calculations are more likely to yield invalid trajectories when the active potential is S_3_. However, the discarded trajectories are similar when comparing PGCs included from the start or postprocessed AXE and SXE trajectories. As a result, the electronic and nuclear dynamics captured by the remaining trajectories are expected to be the same for all three methods, allowing comparisons of the respective observables.

**Table 2:** Number of Trajectories Initially Run, Valid after Propagation, and Valid after Balancing for the Simulations Discussed in Section 3

Pulse	Inclusion of PGCs	Initially run	Valid	Balanced
0.65 fs along *e⃗*_*z*_ case	FP	2000	1964	1929
two-state	RP-AXE	3979	3915	3844
	RP-SXE	2000	1947	1883
0.65 fs along *u⃗*	FP	2000	1881	1543
three-state case	RP-AXE	5768	5192	4088
	RP-SXE	2002	1567	1540

## Supplementary Material



## Appendix A

### From the AXE to the SXE

In this section of the appendix, we discuss in detail how the stochastically Selected eXcited-state Ensemble (SXE) can be derived from the All eXcited-state Ensemble (AXE), and show that the expectation values computed based on the stochastic approach, eq 17, converge to the associated expectation values based on AXE trajectories, eq 15, for a sufficiently large stochastic sample. First, the number of initial conditions from the AXE can be reduced by stochastically selecting those ones that are more likely to be excited based on the pump-generated populations, $|{\mathrm{c̃}}_{a}^{\left(g\right)}\left(0\right){|}^{2}$ . This is achieved by introducing the stochastic variable

25
$$h_{g,a}=\{\begin{array}{ll} 1 & \mathrm{with}\;\mathrm{probability}{\;p}_{g,a},\\ 0 & \mathrm{with}\;\mathrm{probability}\;1-p_{g,a} \end{array}$$

where

26
$$p_{g,a}=\frac{{|{\mathrm{c̃}}_{a}^{\left(g\right)}\left(0\right)|}^{2}}{N}$$

with $N\geq \underset{g,a}{\max}\left\{|{\mathrm{c̃}}_{a}^{\left(g\right)}\left(0\right){|}^{2}\right\}$ , ensuring that *p*_*g,a*_ ≤ 1 and such that the mean value of the stochastic variable *h*_*g,a*_ is $E\left(h_{g,a}\right)=p_{g,a}$ . The expectation value of the operator *Â* then reads

27
$$\left\langle {Â}_{\mathrm{SXE}‐h}\left(t\right)\right\rangle =\frac{1}{N_{g}}\overset{N_{g}}{\underset{g=1}{\sum }}\overset{N_{s}-1}{\underset{a=1}{\sum }}Nh_{g,a}\left\langle \Psi^{\left(g,a\right)}\left(t\right)\left|Â\right|\Psi^{\left(g,a\right)}\left(t\right)\right\rangle$$

where SXE-*h* refers to the fact that the stochastic selection relies on the variable *h*_*g,a*_. The mean value of ⟨*Â*_SXE‑*h*_⟩ then reduces to

28
$$\begin{array}{ll} E\left[\left\langle {Â}_{\mathrm{SXE}‐h}\left(t\right)\right\rangle \right] & =\frac{1}{N_{g}}\overset{N_{g}}{\underset{g=1}{\sum }}\overset{N_{s}-1}{\underset{a=1}{\sum }}{|{\mathrm{c̃}}_{a}^{\left(g\right)}\left(0\right)|}^{2}\\ & \times \left\langle \Psi^{\left(g,a\right)}\left(t\right)\left|Â\right|\Psi^{\left(g,a\right)}\left(t\right)\right\rangle \end{array}$$

which corresponds to eq 15. Evaluating *h*_*g,a*_ in eq 27 yields an ensemble only running over those *N*_t_ selected initial conditions *j* = (*g*,*a*) ∈ S for which *h*_*g,a*_ = 1. The expectation value then reads

29
$$\left\langle {Â}_{\mathrm{SXE}‐h}\left(t\right)\right\rangle =\frac{N\alpha }{N_{t}}\underset{\left(g,a\right)\in S}{\sum }\left\langle \Psi^{\left(g,a\right)}\left(t\right)\left|Â\right|\Psi^{\left(g,a\right)}\left(t\right)\right\rangle$$

where we have also defined $\alpha =\underset{N_{g}\to \infty }{\lim}\left\{N_{t}/N_{g}\right\}$ , which quantifies how many initial conditions (*g*,*a*) are selected for a sufficiently large number of geometries in the AXE. However, eq 29 indirectly depends on the initial populations $|{\mathrm{c̃}}_{a}^{\left(g\right)}\left(0\right){|}^{2}$ in that any new change of the pulse characteristics would require that one repeats the stochastic selection of the initial conditions and all subsequent calculations.

We circumvent this disadvantage by generating an ensemble that is independent of the pump-generated populations but rather depends on steady-state properties, such as, e.g., the oscillator strengths $f_{0a}^{\left(g\right)}$ . This requires the definition of different stochastic variables,

30
$${\mathrm{h̅}}_{g,a}=\{\begin{array}{ll} 1 & \mathrm{with}\;\mathrm{probability}{\;\mathrm{p̅}}_{g,a},\\ 0 & \mathrm{with}\;\mathrm{probability}\;1-{\mathrm{p̅}}_{g,a} \end{array}$$

where *p̅*_*g,a*_ is now defined according to eq 16, with $\mathrm{N̅}\geq \underset{g,a}{\max}\left\{f_{0a}^{\left(g\right)}\right\}$ ensuring that *p̅*_*g,a*_ ≤ 1. In analogy to eq 27 but introducing *h̅*_*g,a*_ for which $E\left[{\mathrm{h̅}}_{g,a}\right]={\mathrm{p̅}}_{g,a}$ , we find

31
$$\begin{array}{ll} \left\langle {Â}_{\mathrm{SXE}‐\mathrm{h̅}}\left(t\right)\right\rangle & =\frac{1}{N_{g}}\overset{N_{g}}{\underset{g=1}{\sum }}\overset{N_{s}-1}{\underset{a=1}{\sum }}\frac{{|{\mathrm{c̃}}_{a}^{\left(g\right)}\left(0\right)|}^{2}}{{\mathrm{p̅}}_{g,a}}{\mathrm{h̅}}_{g,a}\\ & \times \left\langle \Psi^{\left(g,a\right)}\left(t\right)\left|Â\right|\Psi^{\left(g,a\right)}\left(t\right)\right\rangle \end{array}$$

where SXE-*h̅* refers to the fact that the variable *h̅*_*g,a*_ was used, such that also in this case, the mean value $E\left[\left\langle {Â}_{\mathrm{SXE}-\mathrm{h̅}}\left(t\right)\right\rangle \right]$ agrees with eq 15. Finally, replacing *p̅*_*g,a*_ with its definition and evaluating the stochastic variables *h̅*_*g,a*_, retaining only the *N̅*_t_ initial conditions *j* = (*g*,*a*) corresponding to *h̅*_*g,a*_ = 1, we find eq 17, with the properly defined $\mathrm{\alpha ̅}=\underset{N_{g}\to \infty }{\lim}\left\{{\mathrm{N̅}}_{t}/N_{g}\right\}$ . In the rest of this work, the subscript SXE refers to the subscript SXE-*h̅* defined here.

## Appendix B

### The SXE for Multiple Energy Ranges

The SXE leads, by construction, to unequally represented initial active states when the excited states have very different oscillator strengths. In the context of steady-state spectroscopy simulations and corresponding femtochemistry studies, it is an advantage as it drastically reduces the number of initial conditions required for the less absorbing states, compared to the bright ones, which are more important for the absorption spectrum. However, when a pulse is designed to re-balance the importance between dark and bright states, such as, e.g., in the three-state case presented in Section 3.4, the use of the initial SXE leads to very slow convergence. As mentioned in Section 3.4, to speed up the convergence, we split the energy range of interest into *N*_r_ different energy ranges *I*, [*ω*_*I*,lower_,*ω*_*I*,upper_]. The expectation values are obtained exactly in the same way as Equation 17, only summing the contributions from the ensemble associated with different energy ranges *I*, with the different SXE ${\mathrm{S̅}}_{I}$

32
$$\begin{array}{ll} \left\langle {Â}_{\mathrm{SXE}}\left(t\right)\right\rangle & =\overset{N_{r}}{\underset{I=1}{\sum }}\underset{\left(g_{I},a_{I}\right)\in \overset{®}{S_{I}\;}}{\sum }\frac{{\mathrm{\alpha ̅}}_{I}}{{\mathrm{N̅}}_{t,I}}\frac{{|{\mathrm{c̃}}_{a_{I}}^{\left(g_{I}\right)}\left(0\right)|}^{2}}{{\mathrm{p̅}}_{a_{I}}^{\left(g_{I}\right)}}\\ & \times \left\langle \Psi^{\left(g_{I},a_{I}\right)}\left(t\right)\left|Â\right|\Psi^{\left(g_{I},a_{I}\right)}\left(t\right)\right\rangle \end{array}$$

where ${\mathrm{p̅}}_{a_{I}}^{\left(g_{I}\right)}=f_{0a_{I}}^{\left(g_{I}\right)}/{\mathrm{N̅}}_{I}=f_{0a}^{\left(g\right)}/{\mathrm{N̅}}_{I}$ . In analogy to the notations of Table 1, eq 32 can be further recast as in eq 22.

The energy ranges *I* are chosen so as to generate a reasonably biased total sample, suitable for post-processing the effect of the pulse by re-propagating the electronic coefficients along the SXE trajectories for each energy range

33
$$\begin{array}{ll} \left\langle {Â}_{\mathrm{RP}‐\mathrm{SXE}}\left(t\right)\right\rangle & =\overset{N_{r}}{\underset{I=1}{\sum }}\underset{\left(g_{I},a_{I}\right)\in \overset{®}{S_{I}\;}}{\sum }\frac{{\mathrm{\alpha ̅}}_{I}}{{\mathrm{N̅}}_{t,I}}\frac{{|{\mathrm{c̃}}_{a_{I}}^{\left(g_{I}\right)}\left(0\right)|}^{2}}{{\mathrm{p̅}}_{a_{I}}^{\left(g_{I}\right)}}\\ & \times \left\langle \Phi^{\left(g_{I},a_{I}\right)}\left(t\right)\left|Â\right|\Phi^{\left(g_{I},a_{I}\right)}\left(t\right)\right\rangle \end{array}$$

## Appendix C

### Re-Scaling of the Initial Excitation

The total and individual expectation values outlined in eq 11, eq 18 and eq 21, in the regime of weak electric fields, are the physical quantities of interest but are expected to be weak contributions to a small total observable. It is therefore more convenient to look at the individual expectation values for the normalized wave functions. For visualization purposes, however, it is useful to compare the resulting expectation values averaged over the complete ensemble with the expectation values of the individual trajectories. This can be obtained by performing weighted averages rather than simple averages. In the following, we summarize the equations used to produce the results presented in Section 3.

For PGCs included from the start, the expectation value in eq 11 can be written as

34
$$\left\langle {Â}_{\mathrm{FP}}\left(t\right)\right\rangle =\overset{N_{g}}{\underset{g=1}{\sum }}w_{g}\left\langle \Psi^{\left(g\right)}\left(t\right)\left|Â\right|\Psi^{\left(g\right)}\left(t\right)\right\rangle$$

where we have introduced the geometry-dependent coefficients

35
$$w_{g}=\frac{1}{N_{g}}\overset{N_{s}-1}{\underset{k=1}{\sum }}{|{\mathrm{c̃}}_{k}^{\left(g\right)}\left(0\right)|}^{2}$$

By interpreting the coefficients *w*_*g*_ as the weights of a weighted average, one can recast eq 34 into a weighted average given by

36
$$\left\langle {Â}_{\mathrm{FP},w}\left(t\right)\right\rangle =\frac{\overset{N_{g}}{\underset{g=1}{\sum }}w_{g}\left\langle \Psi^{\left(g\right)}\left(t\right)\left|Â\right|\Psi^{\left(g\right)}\left(t\right)\right\rangle }{\overset{N_{g}}{\underset{g=1}{\sum }}w_{g}}$$

Analogously, the expectation values obtained by re-propagating along the AXE trajectories, eq 18, can be written as

37
$$\left\langle {Â}_{\mathrm{RP}‐\mathrm{AXE}}\left(t\right)\right\rangle =\overset{N_{g}}{\underset{g=1}{\sum }}\overset{N_{s}-1}{\underset{a=1}{\sum }}w_{g,a}\left\langle \Phi^{\left(g,a\right)}\left(t\right)\left|Â\right|\Phi^{\left(g,a\right)}\left(t\right)\right\rangle$$

with the geometry- and state-dependent weights

38
$$w_{g,a}=\frac{1}{N_{g}}{|{\mathrm{c̃}}_{a}^{\left(g\right)}\left(0\right)|}^{2}$$

and also in this case, with an associated weighted average given by

39
$$\left\langle {Â}_{\mathrm{RP}‐\mathrm{AXE},w}\left(t\right)\right\rangle =\frac{\overset{N_{g}}{\underset{g=1}{\sum }}\overset{N_{s}-1}{\underset{a=1}{\sum }}w_{g,a}\left\langle \Phi^{\left(g,a\right)}\left(t\right)\left|Â\right|\Phi^{\left(g,a\right)}\left(t\right)\right\rangle }{\overset{N_{g}}{\underset{g=1}{\sum }}\overset{N_{s}-1}{\underset{a=1}{\sum }}w_{g,a}}$$

The denominators in both weighted averages are identical,

(AXE)∑g=1Ng∑a=1Ns−1wg,a=∑g=1Ng∑a=1Ns−11Ng|c̃a(g)(0)|2=∑g=1Ng1Ng(∑a=1Ns−1|c̃a(g)(0)|2)=∑g=1Ngwg⁣(FP)
40

so that comparing results obtained by the weighted averages in eq 36 and eq 39 is equivalent to comparing the corresponding simple averages in eq 11 and eq 18.

For the re-propagated electron dynamics along the SXE trajectories, the same reasoning can be followed,

41
$$\left\langle {Â}_{\mathrm{RP}‐\mathrm{SXE}}\left(t\right)\right\rangle =\underset{\left(g,a\right)\in \mathrm{S̅}}{\sum }w_{g,a}\left\langle \Phi^{\left(g,a\right)}\left(t\right)\left|Â\right|\Phi^{\left(g,a\right)}\left(t\right)\right\rangle$$

with the geometry- and state-dependent weights

42
$$w_{g,a}=\frac{\mathrm{N̅}\mathrm{\alpha ̅}}{{\mathrm{N̅}}_{t}}\frac{{|{\mathrm{c̃}}_{a}^{\left(g\right)}\left(0\right)|}^{2}}{f_{0a}^{\left(g\right)}}$$

The associated weighted average is then given by

43
$$\left\langle {Â}_{\mathrm{RP}‐\mathrm{SXE},w}\left(t\right)\right\rangle =\frac{\underset{\left(g,a\right)\in \mathrm{S̅}}{\sum }w_{g,a}\left\langle \Phi^{\left(g,a\right)}\left(t\right)\left|Â\right|\Phi^{\left(g,a\right)}\left(t\right)\right\rangle }{\underset{\left(g,a\right)\in \mathrm{S̅}}{\sum }w_{g,a}}$$

Using the stochastic variables *h̅*_*g,a*_, one could show that the denominator for the SXE fully agrees with the one for the AXE, eq 40. Comparing eq 18 and eq 21 is therefore fully equivalent to directly comparing eq 37 and eq 41.

Such a weighted average can also be performed for the multiple ensemble variant of the SXE trajectories, eq 33 in Appendix B, defining the ensemble-, geometry-, and state-dependent weights

44
$$w_{I,g,a}=\frac{{\mathrm{N̅}}_{I}{\mathrm{\alpha ̅}}_{I}}{{\mathrm{N̅}}_{t,I}}\frac{{|{\mathrm{c̃}}_{a}^{\left(g\right)}\left(0\right)|}^{2}}{f_{0a}^{\left(g\right)}}$$

The associated weighted average is then given by

45
$$\left\langle {Â}_{\mathrm{RP}‐\mathrm{SXE},w}\left(t\right)\right\rangle =\frac{\overset{N_{r}}{\underset{I=1}{\sum }}\underset{\left(g,a\right)\in {\mathrm{S̅}}_{I}}{\sum }w_{I,g,a}\left\langle \Phi^{\left(g,a\right)}\left(t\right)\left|Â\right|\Phi^{\left(g,a\right)}\left(t\right)\right\rangle }{\overset{N_{r}}{\underset{I=1}{\sum }}\underset{\left(g,a\right)\in {\mathrm{S̅}}_{I}}{\sum }w_{I,g,a}}$$

## Appendix D

### Choice of the Active States in the AXE

In Section 2, all equations regarding the averages with the AXE, including the weighted average in eq 39, were derived without specifying how many states *N*_s_ are selected. Numerically, our implementation relies on a deterministic choice of which electronic excited states for each geometry ***R*** ^(*g*)^(0) are *worth* including as active states in the AXE initial conditions (*g*, *a*). An excited state is namely selected as active if its oscillator strength, limited to the direction of the pulse used, represents more than 1% of the total oscillator strength for the considered geometry. In other words, the electronic states whose contributions to the absorption spectrum in the direction of the target pump pulse are negligible are not considered as active states and the associated initial conditions are not propagated. In practice, this implies that, instead of running *N*_s_ – 1 = 4 trajectories for each geometry ***R*** ^(*g*)^(0) for the presented glycine study, only *N*_ran_(*e⃗*_*z*_) ≃ 2 and *N*_ran_(*u⃗*) ≃ 3 were run for the two-state and three-state cases, respectively.

This can be understood as truncating (or pruning, depending on the order of the selected *N*_ran_ < *N*_s_ – 1 states) the expectation value ⟨*Â*_RP‑AXE_(*t*)⟩ in eq 18; or as introducing an intermediate normalization in the weighted-averaged expectation value ⟨*Â*_RP‑AXE, *w*_(*t*)⟩ in eq 39.

## Notes

The distribution of electronic coefficients in the 1.15 and 0.65 fs pulse situations in the two-state case; PGCs included from the start or postprocessed from the AXE and SXE trajectories for the nonadiabatic dynamics initialized with the ad hoc pulse and 1.15 fs pulse situations in the two-state case; the distribution of the correcting weights in the SXE for the 1.15 and 0.65 fs pulse situations in the two-state case; PGCs included from the start with TSH-EDC or postprocessed with TSH-PFM for the AXE or SXE trajectories precomputed with TSH-EDC; the dipole strengths and components from PGCs included from the start or postprocessed from the AXE or SXE trajectories with the 0.65 fs pulse situation in the three-state case; the distribution of hops in time for the FP approach and AXE trajectories in the three-state case; the state-average molecular orbitals of the CAS­(6,4)-SA5 for the glycine molecule (PDF)
