# Supplementary material for: Accounting for Electronic Coherences Induced by Broadband Pulses by Using Pulse-Independent Trajectories
Source: J Chem Theory Comput. 2026 Jan 20;22(3):1224–43. doi: 10.1021/acs.jctc.5c01809 (PMC12895409; doi:10.1021/acs.jctc.5c01809)
Supplement: Supplementary file 1 [file ct5c01809_si_001.pdf]

Supporting Information to: Accounting for electronic coherences induced by broadband pulses by using pulse-independent trajectories

Joachim Galiana,[*,†] Stefano M. Cavaletto,[*,†] Gilbert Grell,[‡,†] Francisco Fernández-Villoria,[‡,†] Alicia Palacios,[†,¶] Jesús González-Vázquez,[†] and Fernando Martín[*,†,‡]

†*Departamento de Química, Universidad Autónoma de Madrid, Madrid 28049, Spain*

‡*Instituto Madrileño de Estudios Avanzados en Nanociencia (IMDEA Nanociencia), Madrid 28049, Spain*

¶*Instituto de Física de la Materia Condensada (IFIMAC), Universidad Autónoma de Madrid, Madrid 28049, Spain*

E-mail: joachim.galiana@uam.es; stefano.cavaletto@uam.es; fernando.martin@uam.es

Contents

1 Initial Pump-generated Coherences **S2**

2 TSH-PFM with 1.15-fs and Ad Hoc Pulses **S3**

3 Note on the Convergence of the RP-SXE **S5**

4 TSH-EDC Calculations and Post-processing **S6**

5 Dipoles in the Three-state Case **S10**

6 Time Distribution of Hops in the Three-state Case **S12**

7 Active Space for CASSCF Calculations **S12**

1 Initial Pump-generated Coherences

The distributions of the initial electronic coefficients in the presence of the 1.15-fs and 0.65-fs pulse along the $\vec{e}_z$ direction are displayed in Figure S1. Consistently with the population difference or the coherence discussed in the main text, one can observe that the $c_i^{(g)}(0)$ distributions are more uniformly spread in the case of the 0.65-fs pulse, while the 1.15-fs pulse generates 1:0 or 0:1 situtations, with respect to the S_1 and S_2 states, for about 30% of the sample size.

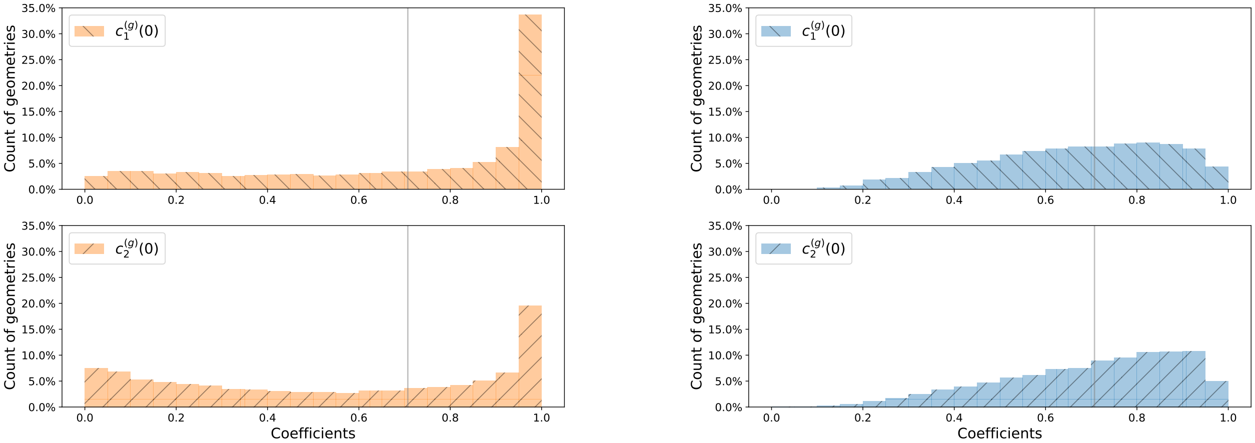

Figure S1. Histograms of the initial coefficients for the first two electronic excited states S_1 and S_2 for the initial Wigner sampling interacting with the 1.15-fs and 0.65-fs Gaussian pulses, left and right, respectively. The vertical gray lines correspond to the ideal but ad hoc pulse exciting both states equally, with maximum initial coherence.

In both cases, displaying $c_2^{(g)}(0)$ as a function of $c_1^{(g)}(0)$, see Figure S2, illustrates how close the pulse-induced superpositions are to an ideal two-state scenario.

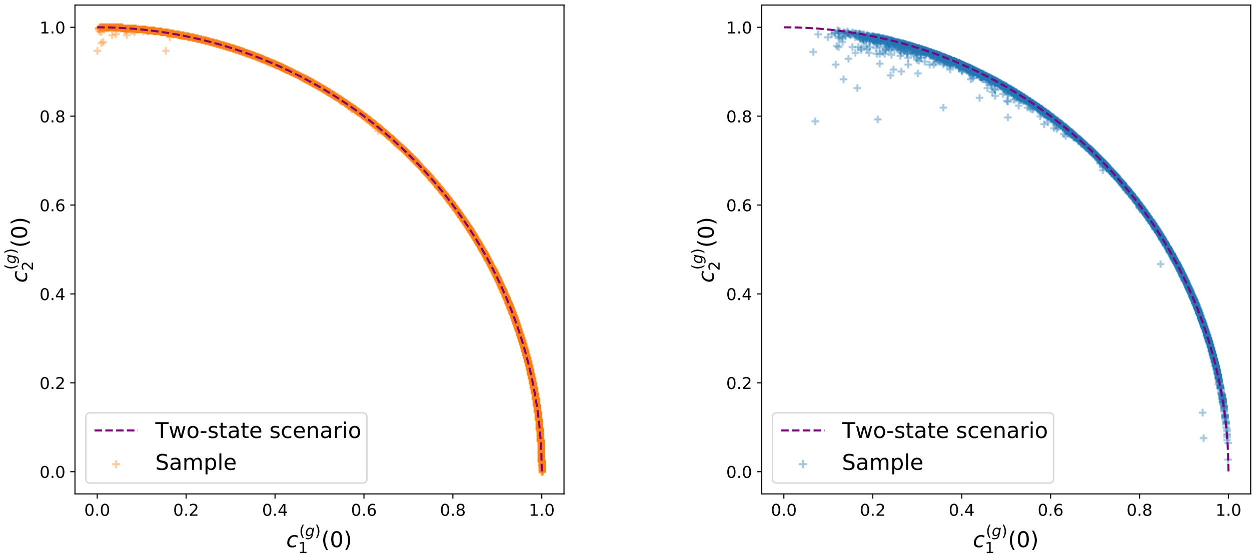

Figure S2. Initial coefficients of the second electronic excited state S_2 and as a function of the ones of the first electronic excited state S_1, for the initial Wigner sampling interacting with the 1.15-fs and 0.65-fs Gaussian pulses, left and right, respectively. The purple dashed lines correspond to the ideal and exact two-state scenario, satisfying $|c_1|^2 + |c_2|^2 = 1$.

2 TSH-PFM with 1.15-fs and Ad Hoc Pulses

The comparisons between full propagation (FP) of the PGCs and re-propagation of the PGCs along the AXE and SXE trajectories (RP-AXE and RP-SXE), extensively discussed in the main text for the 0.65-fs pulse, are here displayed for the ad hoc pulse and the 1.15-fs pulse, Figures S3 and S4, respectively. Similar conclusions are drawn for the three pulses polarized along the $\vec{e}_z$ direction.

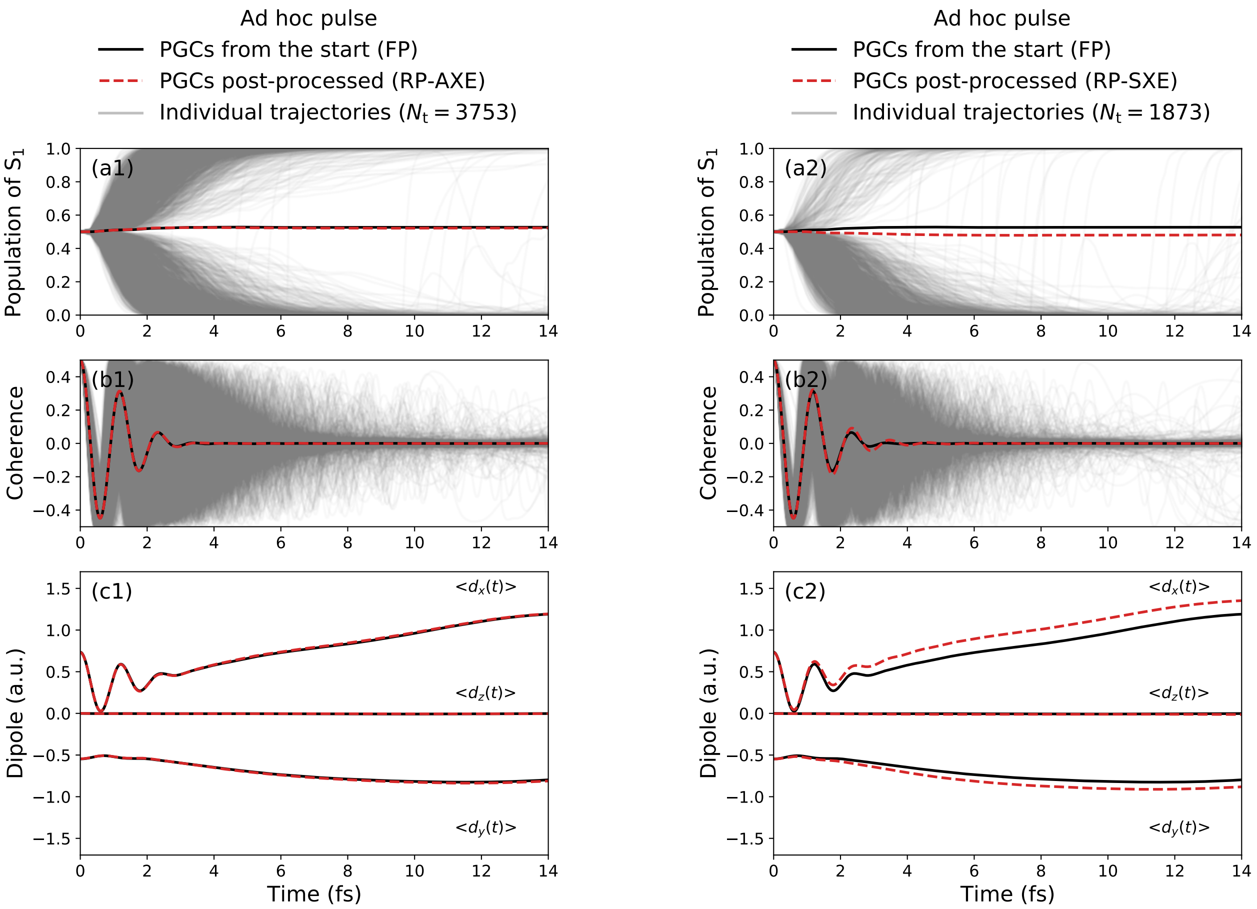

Figure S3. Same as Figure 5 from the main text, but for the ad hoc pulse and the corresponding PGCs.

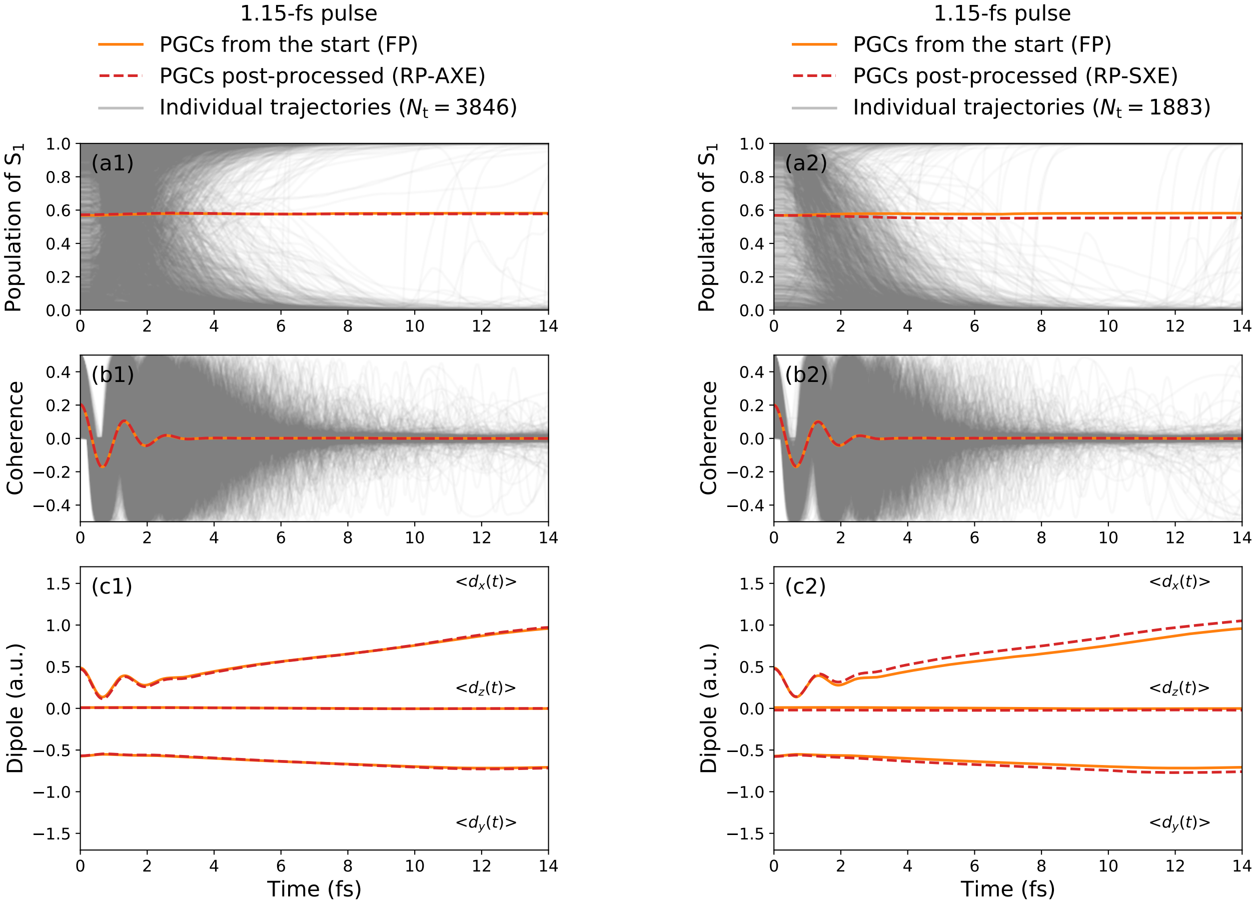

Figure S4. Same as Figure 5 from the main text, but for the 1.15-fs pulse and the corresponding PGCs.

3 Note on the Convergence of the RP-SXE

For the re-propagated PGCs along the SXE trajectories, the geometry- and state-dependent weights

$$w_{g,a} = \frac{\bar{\alpha}}{\bar{N}_\mathrm{t}} \frac{\left|\tilde{c}_a^{(g)}(0)\right|^2}{\bar{p}_a^{(g)}}$$

are used to compute the averaged observables. As discussed in the main text, these weights are built as the ratio of the initial population in the presence of the pulse

$$\left|\tilde{c}_a^{(g)}(0)\right|^2 = \left|c_a^{(g)}(0)\right|^2 \sum_{i=1}^{N_\mathrm{s}-1} \left|\tilde{c}_i^{(g)}(0)\right|^2$$

and the probability of having initially selected the active potential a for the initial condition (g, a), $\bar{p}_a^{(g)}$. This ratio is displayed in Figure S5 for the 1.15-fs and the 0.65-fs pulses, where the blue, orange, and green points indicate trajectories initialized in S_1, S_2, and S_3, respectively. One can notice the important number of selected conditions for S_2, but the relatively stronger weights given to S_1 compared to S_2, which causes the approximately 50:50 population of both states on average.

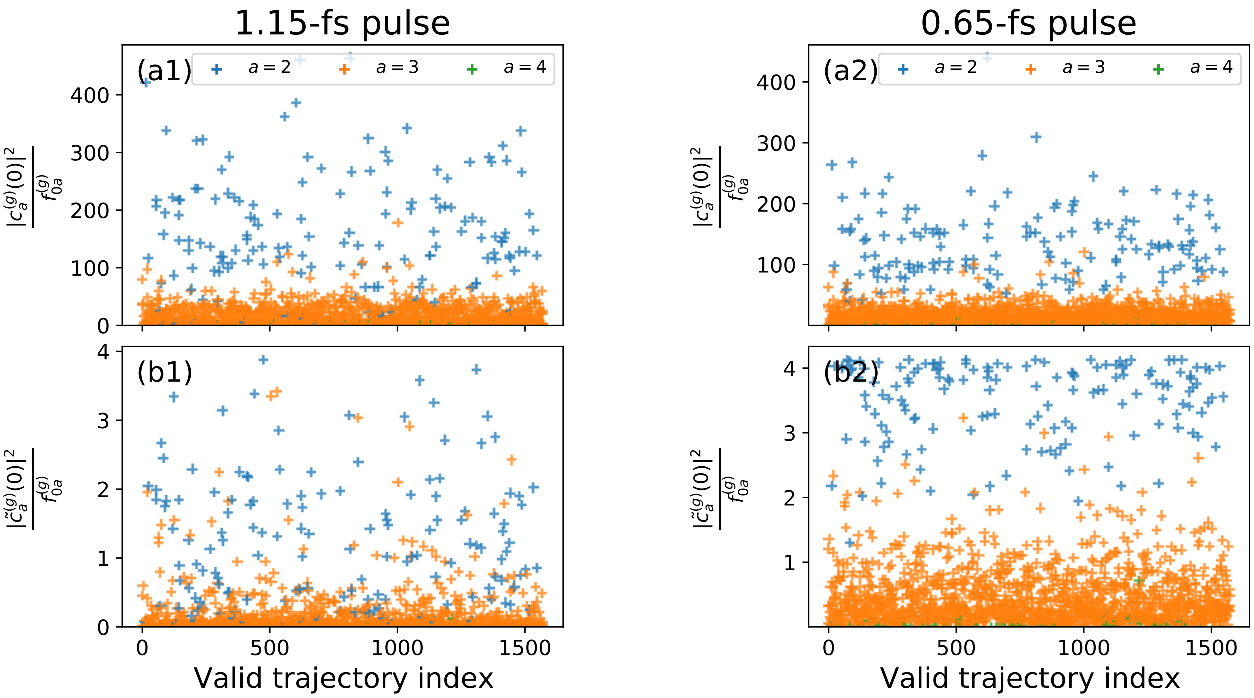

Figure S5. Panels (a1, a2), weights assigned to trajectories initialized in potential active state $a = 2, 3, 4$ (S_1, S_2, and S_3, in blue, orange, and green, respectively). Panels (b1, b2), same re-scaled with the total absorption probabilities for a given geometry, $\sum_{i=1}^{N_s-1} |\tilde{c}_i^{(g)}(0)|^2$. Left and right panels correspond to 1.15-fs and 0.65-fs pulses, respectively.

4 TSH-EDC Calculations and Post-processing

The nonadiabatic dynamics resulting from the full propagation with TSH-EDC (FP, EDC) of the pump-generated coherences (PGCs) associated with the 0.65-fs pulse is displayed in Figure S6, and is to be compared with Figure 4 from the main text (FP, PFM). The electronic coherence on the individual-trajectory level is damped in a stronger way compared to TSH-

PFM, as can be seen from the rapid changes of the individual populations in Figure S6(a) or the smaller overall coherence, Figure S6(b). This is also reflected in the average behavior of both the population and coherence.

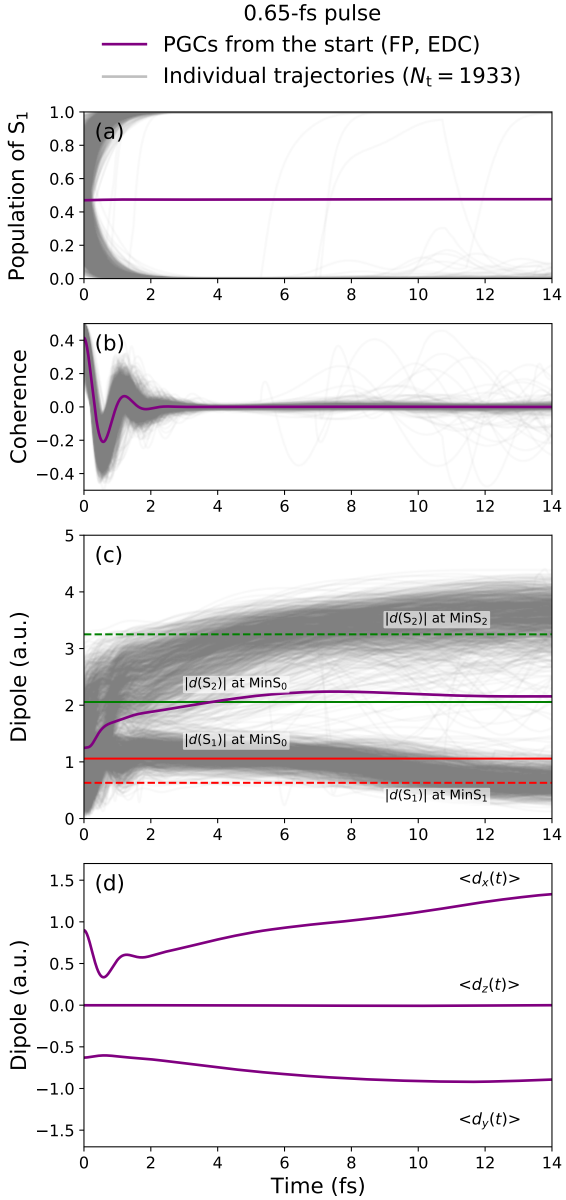

Figure S6. Nonadiabatic dynamics obtained with the FP approach with the EDC scheme, following the excitation of an initial coherent superposition with a 0.65-fs pulse in the presented glycine model. For all panels except (d), both the individual quantities for each trajectory (gray lines) and the total expectation values (purple line) are given. Panel (a): Population $P_1(t) = |c_1(t)|^2$ of the first electronic excited state S_1. Panel (b): Coherence $\mathrm{Re}\left([c_1(t)]^* c_2(t)\right)$. Panel (c): Molecular dipole strength, with reference horizontal lines showing the permanent dipoles of S_1 (red) and S_2 (green) at optimized geometries of the S_0 state (solid) and of the S_1, S_2 states (dashed). Panel (d): Molecular dipole components along x, y, and z directions.

In Figure S7, the re-propagation of the PGCs is performed along AXE or SXE trajectories pre-computed with TSH-EDC. As a result, the PFM decoherence scheme only applies at the

post-processing stage, i.e., the re-propagation of the PGCs along the frozen, pre-computed TSH-EDC trajectories. This differs from the main text where both the propagation of the pre-computed trajectories and the re-propagation of the electronic dynamics are performed with TSH-PFM. The full propagation with TSH-EDC and the full propagation with TSH-PFM are reproduced in Figure S7 for reference, as the blue solid line and purple solid line, respectively. As mentioned in the main text, the results of the RP-AXE and RP-SXE along TSH-EDC pre-computed trajectories (red dashed lines) are perfectly comparable to the full propagation with TSH-PFM (FP, PFM, blue solid line).

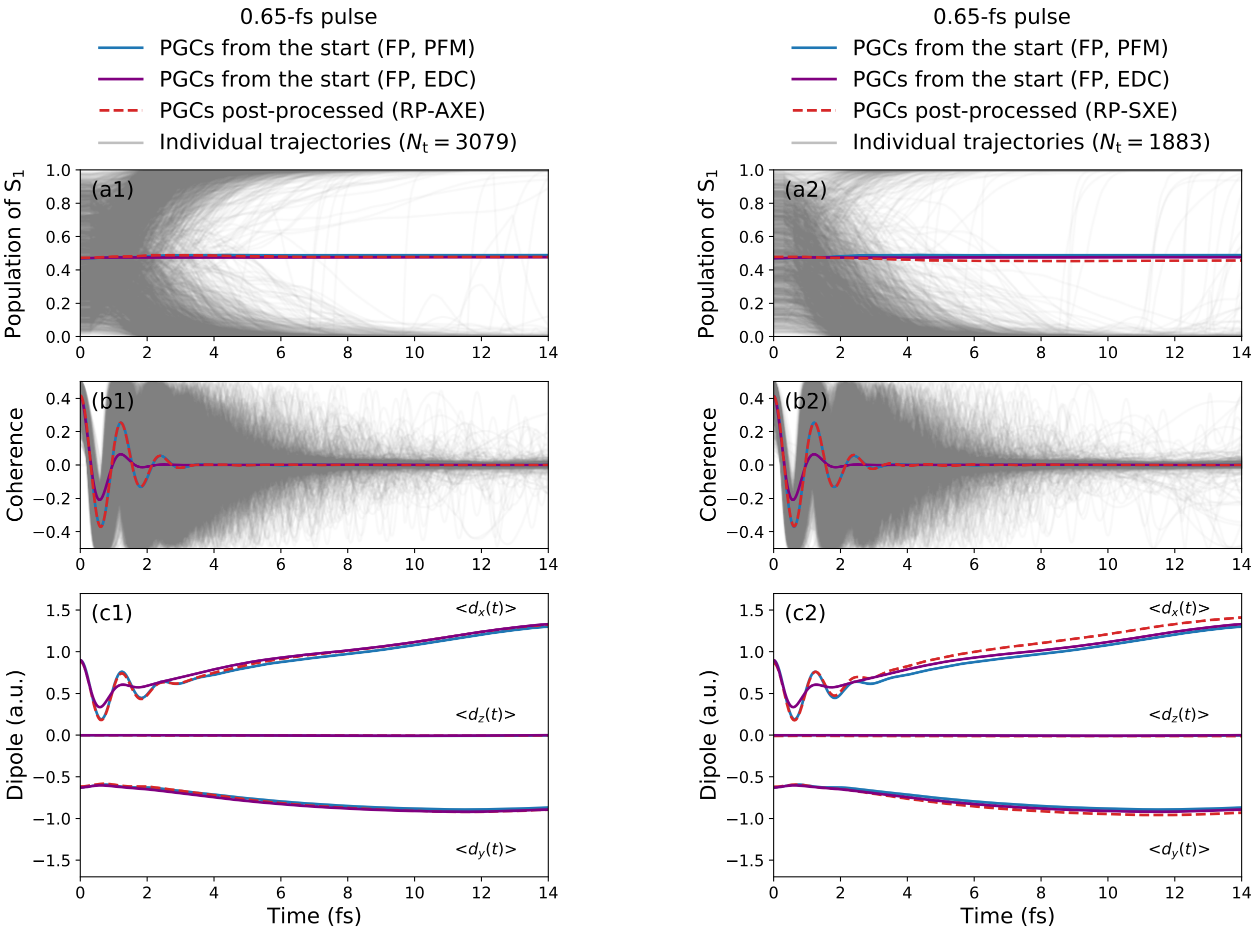

Figure S7. Quantities in panels (a1, a2), (b1, b2), and (c1, c2) are the same as in Figure S6(a), (b), and (d), respectively. The observables obtained by post-processing the PGCs (dashed red lines), with re-propagated electronic coefficients along the AXE and SXE trajectories (gray lines) pre-computed with TSH-EDC, are compared to the observables obtained by including the PGCs from the start with TSH-PFM (solid blue lines) and with TSH-EDC (solid purple lines). The left and right columns correspond to the re-propagation of the PGCs along the AXE and the SXE trajectories, respectively

5 Dipoles in the Three-state Case

For completeness regarding the three-state case discussed in the main text, we display comparison of full propagation (FP) and re-propagated PGCs along AXE and SXE trajectories (RP-AXE and RP-SXE), for the dipole strengths and components, in Figure S8.

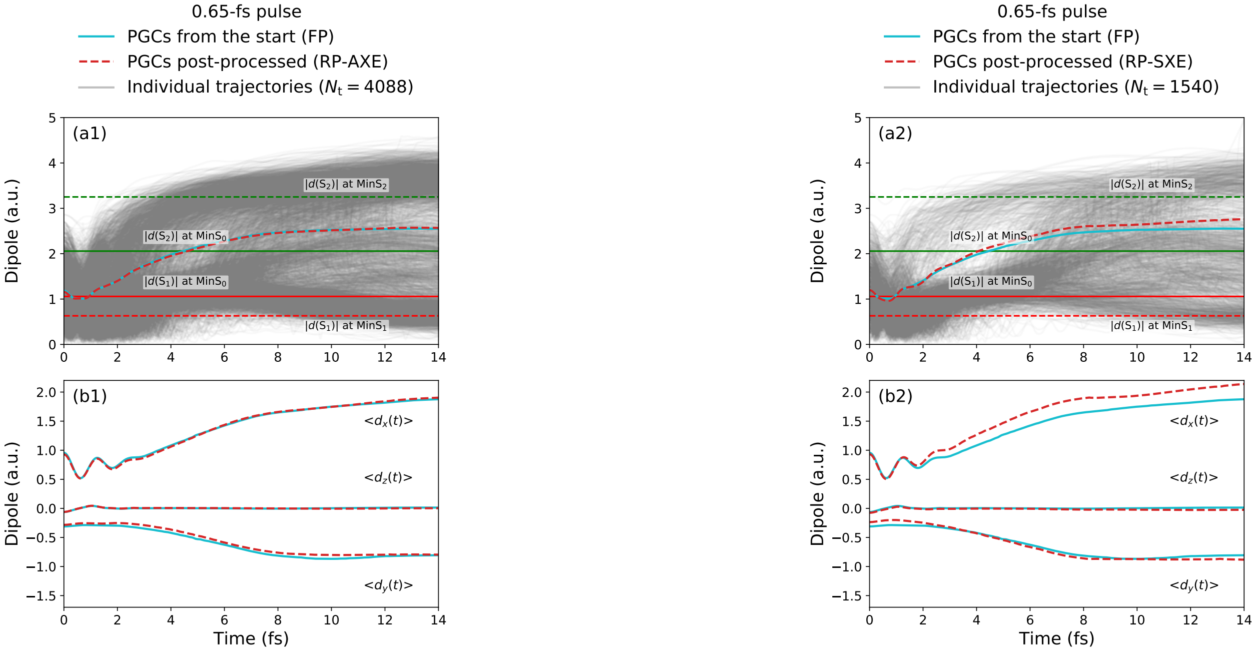

Figure S8. Time evolution of dipole strengths (a1, a2) and x, y, and z components (b1, b2). The observables obtained by post-processing the PGCs (dashed red lines), with re-propagated trajectories (gray lines), are compared to the observables obtained by including the PGCs from the start (solid cyan lines). The left and right columns correspond to the re-propagation of the PGCs along the AXE and the SXE trajectories, respectively.

6 Time Distribution of Hops in the Three-state Case

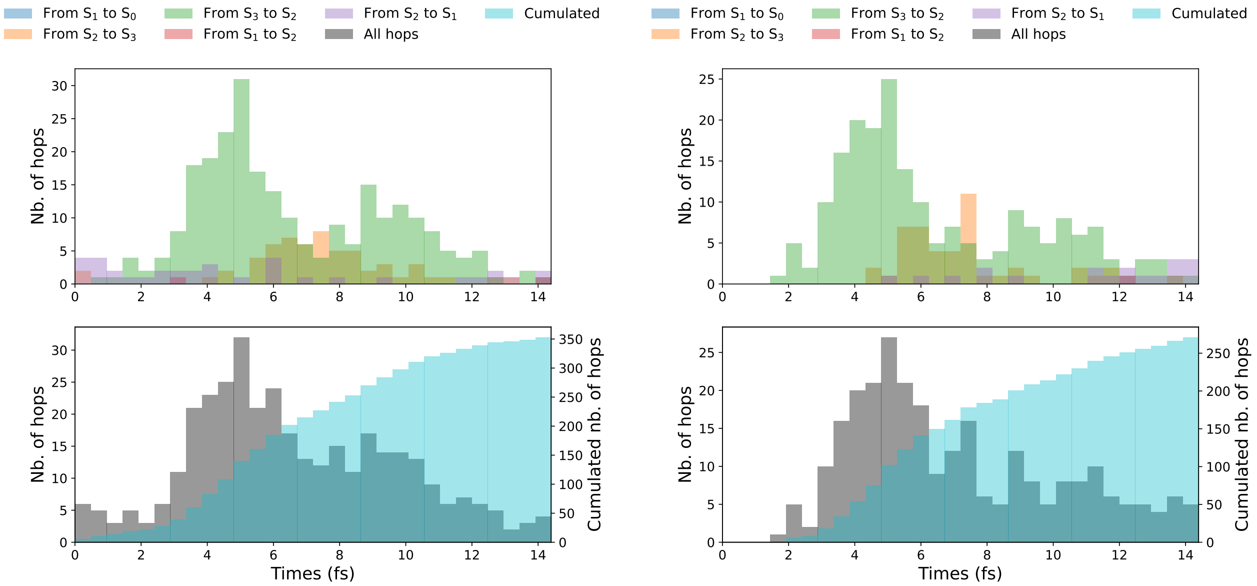

Figure S9. Distribution of the hopping times in the FP and AXE trajectories, in left and right panels, respectively. The top panels display the distributions for some particular cases in colors (from a specific active potential to another); the bottom panels display in gray the distribution of the total number of hops in time (and its cumulated counterpart, in cyan).

7 Active Space for CASSCF Calculations

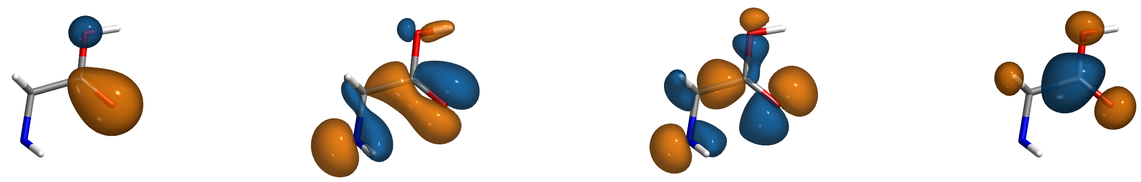

Figure S10. State-average molecular orbitals of glycine at the optimized geometry of the electronic ground state, obtained with CAS(6,4)-SA5 electronic structure calculations using the cc-pVDZ basis set.
